# Supplementary material for: Designing fuel cell catalyst support for superior catalytic activity and low mass-transport resistance
Source: Nat Commun. 2022 Oct 18;13:6157. doi: 10.1038/s41467-022-33892-8 (PMC9579166; doi:10.1038/s41467-022-33892-8)
Supplement: Supplementary file 1 — Supplementary Information [file 41467_2022_33892_MOESM1_ESM.pdf]

Supplementary Information

Designer fuel cell catalyst support for superior catalytic activity and low mass-transport resistance

Muhammad Naoshad Islam[1], Abdul Bashith Mansoor Basha[1], Vinayaraj Ozhukil Kollath[1], Amir Peyman Soleymani[2], Jasna Jankovic[2] and Kunal Karan[*1]

Author Affiliations:

[1] Department of Chemical and Petroleum Engineering, University of Calgary, Calgary, AB, Canada

[2] Center for Clean Energy Engineering, Institute of Materials Science, and Materials Science and Engineering Department, University of Connecticut, Storrs, CT, USA

* Corresponding Author

Experimental

Materials

Dopamine hydrochloride (Milipore-Sigma, Canada), sodium hydroxide (Milipore-Sigma, Canada ACS reagent, ≥ 97.0%, pellets), ethanol (98% purity; Milipore-Sigma, Canada), isopropyl alcohol or IPA (≥ 99.5% purity; Milipore-Sigma, Canada), ethylene glycol (≥ 99.8% purity; Milipore-Sigma, Canada), acetone (ACS Grade, ≥ 99.5%, Fisher Scientific, Canada), hexachloroplatinic acid (H_2PtCl_6, 8 wt% in H_2O, Milipore-Sigma, Canada), TKK 10.2% (TEC10V10E; TKK, Japan) and 19.8% Pt/C (TEC10V20E; TKK, Japan) catalyst (purchased from Fuel Cell Store, USA), Nafion (EW 1100, Ion Power, USA), 70% perchloric acid Veritas doubly distilled (GFS chemicals, USA) were used as received. High-purity de-ionized water (Milli-Q Advantage A10, Millipore SAS) was used.

Synthesis of PDA nanospheres

PDA nanospheres were prepared by a facile one-step aerobic polymerization method reported previously[1]. The monomer (Dopamine hydrochloride, 1 g) was dispersed in DI water in a 20 mL glass vial. A dispersion medium was prepared separately by mixing water-ethanol with 2.1 vol% of (28-30 vol% NH_4OH) in a 500 mL glass beaker. Subsequently, the dopamine dispersion was added slowly to the dispersion medium; here, dopamine concentration was kept constant at 3.3 mg mL^{-1} while maintaining the final water: ethanol ratio at 67:33 vol%. Then, the mixture was stirred for 4 h at 1600 rpm in the presence of air at room temperature (23 ºC). Once polymerized, the sample was centrifuged at 27216 xg (15,000 rpm) (Beckman coulter Avanti J-26S XP, USA) and washed with an ample amount of DI water and ethanol to remove unreacted monomer and other residues. The sample was re-dispersed by sonication and then centrifuged again. The procedure was repeated three times till the dispersion media became pH neutral. The sample was then vacuum dried at 60 ºC. The overall yield (starting monomer to final recovered dry PDA spheres) was calculated to be around 65% (gravimetrically). Carbonization of the PDA nanoparticles

was carried out at 700 ºC under an N_2 atmosphere which yielded smaller-sized but spherical carbonized PDA (cPDA) nanoparticles. The carbonization protocol featured three stages: (i) heating from room temperature to 700 °C with a heating rate of 5 °C/min; (ii) a dwell time of 1 h at 700 °C for complete carbonization; and (iii) slow, uncontrolled cooling to room temperature.

Platinum deposition on cPDA nanospheres

Pt nanoparticles were deposited on cPDA nanospheres by following a modified polyol method[2]; 500 mg of cPDA were added to 140 mL of Ethylene Glycol (EG) in a 400 mL round bottom flask and sonicated for around 30 min, followed by magnetic stirring at 1600 rpm to obtain a well-dispersed slurry. In order to achieve a 10 wt% Pt loading on cPDA, 105 mg of H_2PtCl_6 was dissolved in 35 mL of water. The cPDA dispersion was preheated at 140 ºC in an oil bath, and H_2PtCl_6 (aq) solution was added dropwise to the dispersion. Subsequently, the pH of the mixture was adjusted to around 10-11 by adding a pre-made 5M NaOH (aq) solution. The reaction mixture was stirred at 1600 rpm for approximately 4 hours under reflux. The pH was monitored using a pH meter (Model EL20, Mettler Toledo, USA) during this period and maintained at the desired level (between 10 and 11) by adding the NaOH (aq) solution if required. The level of the solution in the round bottom flask was maintained by the addition of a 20% (v/v) water/EG mixture. After the completion of the reaction, the sample was filtered and washed with DI water, ethanol and acetone, followed by vacuum drying at 60 ºC. Next, the dried sample was reduced under a 10% H_2 in N_2 atmosphere inside a furnace to ensure complete reduction of deposited Pt. Prior to H_2 treatment, the furnace was purged with N_2. Thereafter, the gas was changed to 10% H_2 in N_2, and the temperature was raised to 300 °C at 5 °C min^{-1}, held for 3 h, and afterwards cooled to room temperature under an N_2 atmosphere.

PDA, cPDA and Pt/cPDA characterization

The size and the morphology of PDA and cPDA nanospheres were examined by using Hitachi H-600 (Japan) Scanning Electron Microscopy (SEM), Tecnai F20 200 kV (USA) Transmission Electron Microscope (TEM) and Talos F200X Scanning transmission electron microscope (USA) equipped with a Super-X four silicon drift detectors of energy dispersive spectrometry (Super-X SSD EDS, EDAX). The TEM thin sections (approximately 100 nm) were cut by Leica UCT ultramicrotome setup (Germany) equipped with an Ultra 45°DiATOME knife (USA) from a block prepared by embedding a small piece of the catalyst-coated membrane (CCM) in a 1:1 mixture of trimethylolpropane triglycidyl ether resin (Sigma-Aldrich, USA) and 4,4′-Methylenebis (2-methylcyclohexylamine, Sigma-Aldrich, USA) hardener, polymerized overnight at 60°C. The sections were situated onto multiple 200 mesh Cu/Pd grids. Laboratory-based X-ray photoelectron spectroscopy (XPS) measurements were carried out at room temperature in an ultrahigh vacuum (UHV, 3×10^{-8} Torr) setup using a monochromatized Al Kα (1486.6 eV) excitation and a hemispherical analyzer (Kratos Axis Ultra DLD, UK). The XPS spectra were analyzed using CasaXPS software. All spectra were charge-corrected with respect to the main C1*s* peak of the corresponding sample, which was assigned to have a binding energy of 284.8 eV[3]. The wide survey XPS spectra of PDA, cPDA and Pt/cPDA samples are presented in Fig. S2(a). The high resolution deconvoluted XPS spectra of C1*s*, O1*s* and Pt 4*f* peaks (for Pt/cPDA only) for PDA, cPDA, and Pt/cPDA are shown in Fig. S2(b-d), while the high resolution deconvoluted XPS spectra of N1*s* for PDA, cPDA and Pt/cPDA has been presented in the main manuscript Fig. 2(f).

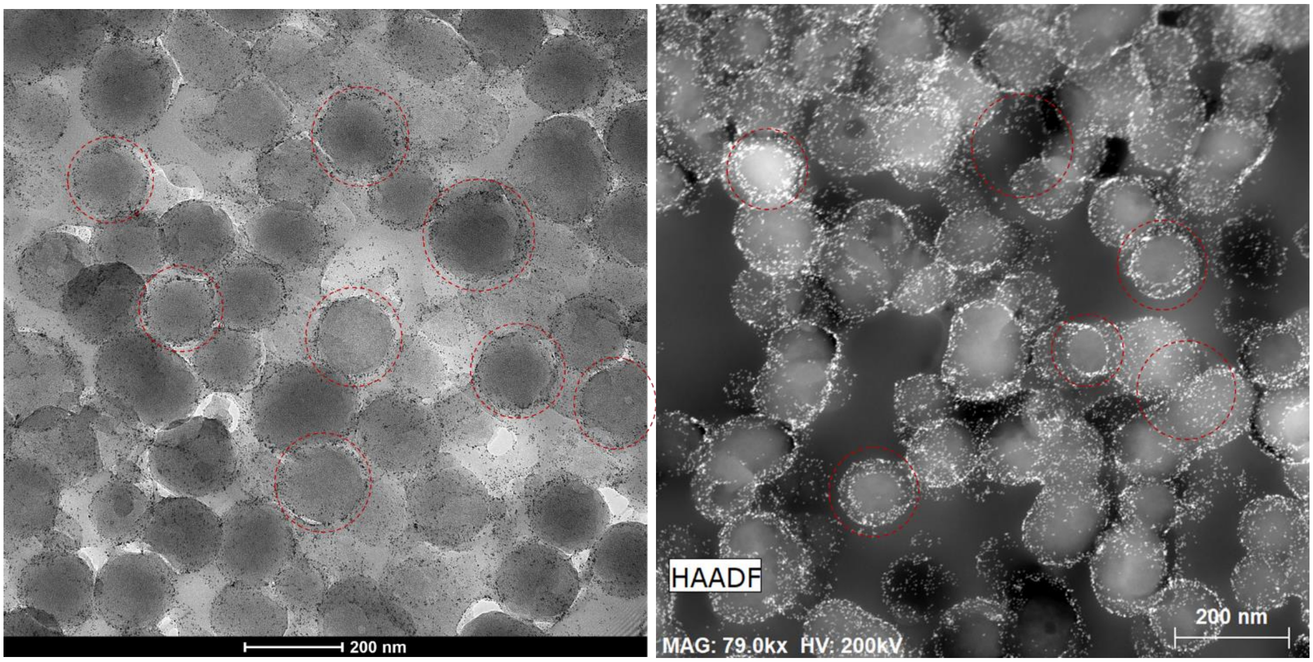

Fig. S1. STEM image of microtomed Pt/cPDA CL. The marked regions are the cross-section of Pt/cPDA catalysts highlighting the location of the Pt particles (left - Brightfield, right - Darkfield).

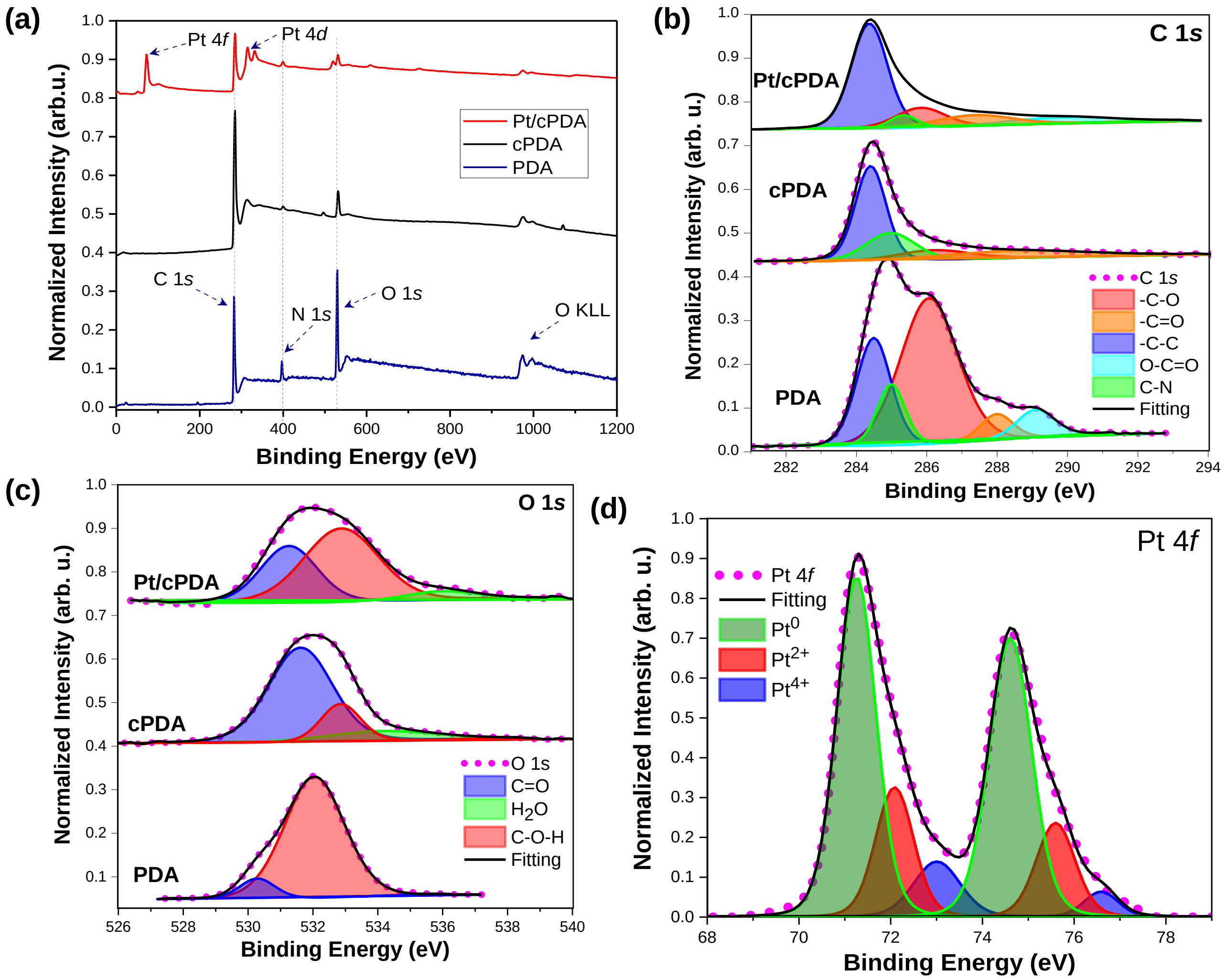

Fig. S2. (a) Wide survey XPS spectra of PDA, cPDA and Pt/cPDA samples; high resolution deconvoluted XPS spectra of (b) C1*s* for PDA, cPDA and Pt/cPDA, (c) O1*s* for PDA, cPDA and Pt/cPDA PDA, (d) Pt 4*f* for Pt/cPDA.

N_2 sorption isotherm

N_2 sorption analysis were performed on PDA and cPDA samples using a 3Flex™ analyzer (USA) (Alberta Sulphur Research Ltd., University of Calgary Research Center). The sample was heated under a vacuum (6-10 mbar) in two stages, initially to 60 °C at 1 °C min^{-1} for 2 h and then to 100 °C at 1 °C min^{-1} for 12 h, following which the out-gassing rate was less than 2 μbar h^{-1}. The sample was then purged with N_2 before being transferred to the analysis port, where it was evacuated for at least a further 240 min before the analysis was started. The temperature was kept constant by using liquid N_2 (76.3 K). The Brunauer Emmet-Teller (BET) model was applied between partial pressures of $0.04 < P/Po < 0.30$ to obtain the specific surface area. The micropore surface area was obtained using a t-plot curve, which was applied between partial pressures of $0.06 < P/Po < 0.7$. Results are summarized in Table S1.

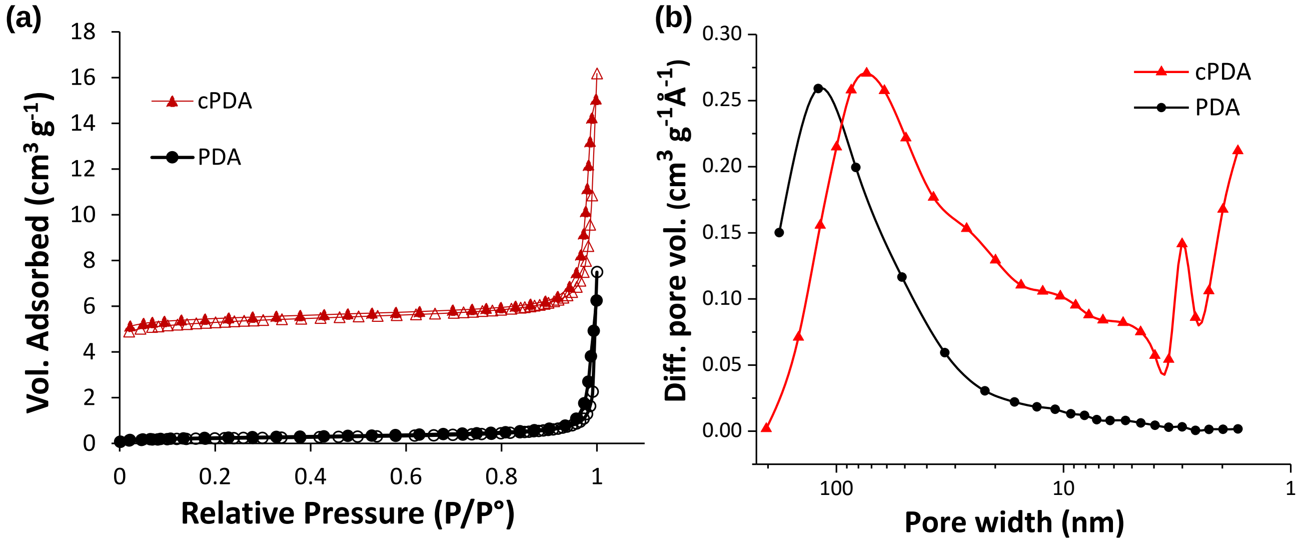

Fig. S3. (a) N_2 adsorption-desorption isotherm data for PDA and cPDA and (b) the corresponding pore size distribution.

Table S1. Microstructural properties of cPDA carbon support and Vulcan[4] carbon determined from N_2 adsorption isotherm.

Sample	$SA_{BET, total}$ $(m^2 g_{carbon}^{-1})$	$SA_{> 2\ nm}$ $(m^2 g_{carbon}^{-1})$	$SA_{< 2\ nm}$ $(m^2 g_{carbon}^{-1})$	$V_{pore, total}$ $(cm^3 g_{carbon}^{-1})$	$V_{pore, < 2nm}$ $(cm^3 g_{carbon}^{-1})$	Ref
cPDA	365.4	62.9	302.5	0.51	0.16	This work
Vulcan carbon	227.7	114.7	113.0	0.4	0.06	[4]

Pt content determination in Pt/cPDA

Pt content of 8.5 wt% was determined by Thermogravimetric analysis (TGA) as described below, and this value was used to calculate mass-specific activity.

Determination of Pt content of catalyst by thermogravimetric analysis (TGA): The Pt content of the catalysts and catalyst layer were determined by thermogravimetric analyses. The set of experiments also yielded ionomer content (ionomer to carbon, I:C ratio) in the Pt/cPDA catalyst layer. The measurements were performed with a Mettler-Toledo TGA/DSC 3+ instrument (USA). In all the TGA experiments, around 4 mg of vacuum dried sample was loaded on a previously tared 70 µL Al_2O_3 TGA crucible and heated from room temperature to 150 °C under N_2 (at 200 mL min^{-1}), followed by a 120 min hold at 150 °C to get rid of any residual moisture content. Prior to use, all the TGA crucibles were cleaned by soaking overnight in aqua regia, followed by 5 min of sonication in DI water and acetone each, and drying at 350 °C (in Air). To oxidize carbon support, the samples were heated in an air environment. To decompose the ionomer only in the Pt/cPDA CL, the sample was heated in a nitrogen environment. A ramp of 10 °C min^{-1} was maintained for all the heating steps.

Pt content of catalyst: Pt content of Pt/C TKK (10.2% and 19.8%) and Pt/cPDA catalysts were determined from the residual weight after oxidation/burning of carbon in an air environment at around 700 °C. First, the samples were dried overnight under a vacuum at 80 °C. Next, the Pt-loaded catalyst supports were heated to 150 °C under N_2 (at 200 mL min^{-1}) to remove any moisture and heated again to oxidize the carbon support (VC-TKK or cPDA support) from 150 °C to around 700 °C under Air (50 mL min^{-1}) until the mass were not changing. This resulted in a Pt content of 8.5% for Pt/cPDA, 19.5% for TKK Pt/C 19.8% and 9.5% for TKK Pt/C 10.2%, respectively (Fig. S4).

Ionomer content in CL: The ionomer and Pt content in Pt/cPDA CL was determined by TGA in two different experiments. Firstly, the ionomer content was determined by decomposing the ionomer only in the CL under N_2. For this, around 4 mg of Pt/cPDA CL was first peeled from the CL decal and dried under a vacuum oven overnight at 80 °C. Then, the dried sample was loaded on a previously cleaned and tared TGA crucible. After that, the sample was heated from room temperature to 150 °C under N_2 (at 200 mL min^{-1}) and held for 120 min to get rid of any residual moisture content. Then, the sample was heated from 150 °C to 700 °C under N_2 (50 mL min^{-1}) to decompose all the ionomer, which yielded an ionomer to carbon ratio (I:C) of 0.75 based on the previously determined Pt content of 8.5%.

Pt content in CL: In a separate experiment, similar to the ionomer content determination experiment, the CL samples were first heated to 150 °C under N_2 (200 mL min^{-1}), followed by heating to 700 °C under Air (50 mL min^{-1}), which oxidized all the carbon support (both cPDA and Vulcan carbon). The Pt content of Pt/cPDA and Pt/C TKK10% CLs was estimated to be around 7.8% and 10.4%, respectively, from the residual weight. All the TGA data are presented in Fig. S4 and Table S2, and Fig. 3c in the main manuscript.

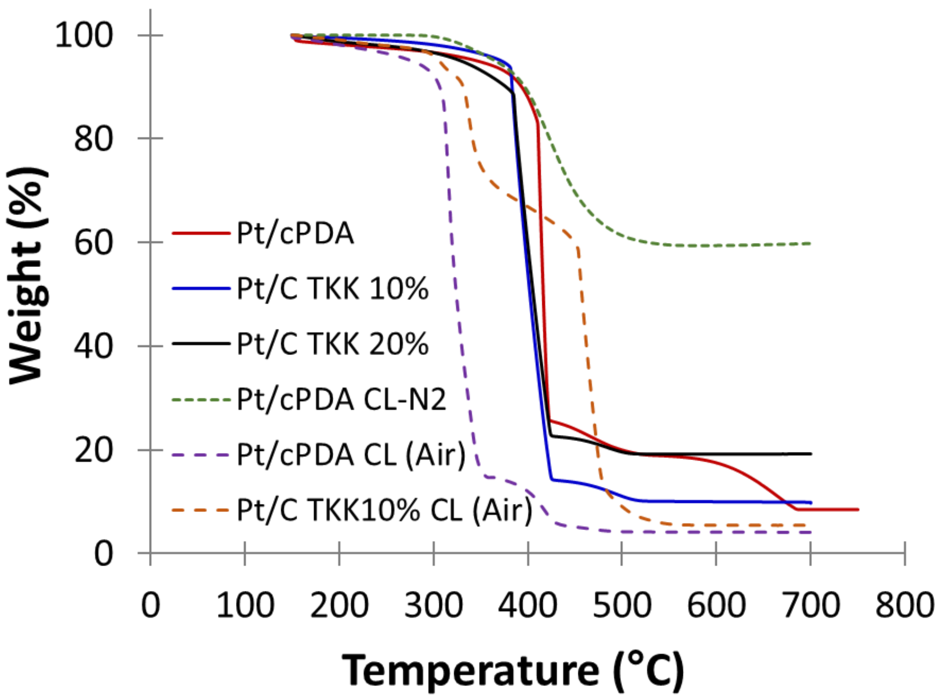

Fig. S4. TGA thermogram of Pt/cPDA, TKK Pt/C 10%, TKK Pt/C 20%, Pt/cPDA CL and TKK Pt/C 10% CL for the determination of Pt and ionomer content (Pt/cPDA CL).

Table S2. Summary of the TGA determined (TGA-Pt/C) and theoretical (Pt/C_{Th}) Pt content in Pt/cPDA CL, TKK 10% Pt/C (Vulcan carbon) CL.

Sample	TGA-Pt/C (%)	Pt/C_{Th} (%)*
Pt/cPDA CL	7.8	-
Pt/cPDA	8.5	
Pt/C TKK 10%	9.5	
Pt/C TKK 10% CL	10.4	10.2
Pt/C TKK 20%	19.5	19.8

*The Pt/C_{Th} for the commercial catalysts are the manufacturer provided values.

Pt content estimation in Pt/cPDA by TEM image analysis: The particles were assumed to be spherical. A total of 173 Pt particles from the TEM image presented in Fig. 3a (main manuscript) were used for this calculation. The surface area (SA) of each particle was calculated as: πD_p^2 and the mass of each particle as a product of the volume of the sphere and density of platinum: $(\pi \times D_p^3/6) \times (\rho_{Pt})$. The number of average ECSA ($\Sigma SA_i/\Sigma m_i$) is 108 $m_{Pt}^2\ g_{Pt}^{-1}$, which is slightly lower than ECSA based on an average particle size of 117 $m_{Pt}^2\ g_{Pt}^{-1}$, where SA_i and m_i are calculated surface area and mass of individual Pt nanoparticles, respectively, assuming that the particles are spherical.

By Dividing ECSA (cm_{Pt}^2/g_{Pt}) by (cm_{Pt}^2/g_{Pt+C}), we obtain the following Pt content on cPDA (Table S3). The differences between TEM, RDE and MEA-based ECSA are commonly observed and are attributed to less than perfect accessibility of Pt catalyst by protons in an MEA or catalyst layer due to ionomer coverage issues. The estimated Pt content of Pt/cPDA catalyst from image-based analyses was calculated to be around 7.4 – 8.5 wt%.

Table S3. Estimated Pt content on cPDA determined from TEM image analysis.

**ECSA Calculation**		**Pt content (wt%); Pt/(Pt+C)**		
Method	$ECSA_{TEM}$ (m^2/g_{Pt})	$Pt/cPDA_{liquid}$ (%)	$Pt/cPDA_{MEA}$ (%)	TGA Pt/cPDA (%)
$\Sigma SA_i/\Sigma m_i$	108	8.4	8.5	8.5%
$\Sigma(SA_i/m_i)$	123	7.3	7.4	
(SA_{avg}/m_{avg})	117	7.7	7.8	

*Assuming all Pt precursor was loaded on cPDA support.

$l_{Pt\text{-}Pt}$ or average inter-particle distance

Average inter-particle distance (AID, nm) was estimated using the equation (S1) developed by Meier et al.[5].

$$\text{Average inter-particle distance (AID)} = \sqrt{\frac{\pi}{3\sqrt{3}}.\,10^{-3}.\,\rho_{Pt}.\left(\frac{100-L_{Pt}}{L_{Pt}}\right).\,A_S.\,d^3} - d \qquad \text{(S1)}$$

Where, ρ_{Pt} is the density of platinum (21.5 g cm^{-3}), L_{Pt} is the platinum content (wt%), A_S is the specific surface area of the support (m^2 g^{-1}), and d is the platinum particle diameter (nm).

Electrochemical Characterization in Liquid Electrolyte

Electrochemical characterization of the Pt/cPDA catalyst and commercial TKK 10% Pt/C catalyst (the same batch as that used for comparison in MEA, see next section in SI) were performed via rotating disk electrode (RDE) test. Extensive care was taken to clean the glassware and work with high purity electrolyte as described in the following sub-section. Significant effort was directed towards the development of in-house expertise to create good quality films out of powdered catalysts based on pertinent key articles ([6–11]) and discussion ([54]).

Rotating Disk Electrode Test

Glassware and component cleaning: The ORR kinetics are incredibly susceptible to trace amounts of impurities even at ppm levels, as is well established in the literature[7,9,18–21,10–17]. Hence, the cleanliness of the electrochemical cell and components is as important as the purity of the electrolyte. Therefore, cleaning all the electrochemical components is obligatory using the following procedure by summarizing the various literature[6–10,12,22–25]. The electrochemical cell glassware (Pine Instrument, USA) and the components were soaked in concentrated sulfuric acid (Millipore Sigma, USA) overnight, followed by a 5-6 times repeat sequence of boiling with DI water followed by DI water replacement after each boil. This procedure is followed after each experiment to eliminate the trace amount of impurities. Before the

electrochemical testing, the electrochemical cell and the components are rinsed 2-3 times with freshly prepared 0.1 M $HClO_4$ (diluted from 70% Veritas Doubly Distilled GFS Chemicals, USA). The glassy carbon (5 mm diameter, Pine Instrument) tips are polished before each experiment using 0.05 µm alumina slurry (Pine Instrument, USA), followed by rinsing and subsequent sonication for 3-5 min using DI water. The glassy carbon (GC) tip was dried using nitrogen gas before the catalyst coating.

Ink formulation and coating: Two different fabrication techniques were applied for the two different catalyst films - (i) ionomer-free and (ii) ionomer-based catalysts based on the approaches described by Shinozaki et.al[7,8,10,11,26] and Kocha et.al[6–9,9–11,27]. For ionomer-free catalyst characterization, the stationary air-dry technique ([6–11]) was followed, whereas the rotational air-dry approach ([6–11]) was adopted for the ionomer-based catalyst film.

The ionomer-free ink was prepared by adding 9.6 mg of catalysts in a 20 vol% IPA in a water mixture. The resulting ink was sonicated in an ice water bath sonicator for ~ 20 min to obtain a good dispersion slurry (10 µl aliquot, ~ 10 μg_{Pt} cm^{-2}). For ionomer-based catalyst, 9.6 mg of catalyst was dispersed in the required amount of 20 vol% IPA in a water mixture to which 39.5 µl of 5 wt% Nafion was further added to achieve an overall I/C mass ratio of 0.2 such that 10 µl aliquot would yield ~ 10 μg_{Pt} cm^{-2} in the coating. The ink was sonicated with an ice water bath sonicator for ~ 20 min. The sonication time of ~ 20 min was found to be optimum; long sonication time or non-ice water bath sonication results in a deleterious effect observed in a loss in ECSA and activity, as is well established in the literature[7,8,11,28]. After sonication, the ink was deposited on the GC tip mounted on the inverted rotator shaft (Pine Instrument, USA) for the ionomer-based catalyst. It was rotated at 500 rpm for 15-20 min until the film dried. An aliquot of ink was deposited onto the GC disk and dried under an air atmosphere for the Nafion-free catalyst. The sample with partial or non-uniform coating assessed by visual inspection was rejected.

Electrochemical characterization in liquid electrolyte: All the electrochemical measurements were carried out using a conventional three-electrode setup consisting of a catalyst film coated on GC as a working electrode, Pt gauze as a counter electrode and a reversible hydrogen electrode (RHE) as a reference electrode.

The electrochemical characterization followed a sequence of experiments (with conditions similar to that employed by other respectable groups) comprising catalyst conditioning, ECSA determination, and ORR activity measurement:

1. The conditioning of the catalyst is performed by cycling the electrode potential from 0.025 V – 1.2 V at 500 mV s^{-1} for 50-100 cycles under 1600 rpm in N_2-saturated 0.1 M $HClO_4$ until repeatable cyclic voltammograms were obtained. The voltage has been restricted to 1.2 V to minimize carbon corrosion. The conditioning of the catalyst is essential to obtain high ORR activity.
2. To evaluate the electrochemical active surface area (ECSA), the H_{UPD} charge was obtained from hydrogen adsorption observed between ~ 0.05 V and ~ 0.4 V in the third cycle of the CV measured in 0.025 V – 1 V at 20 mV s^{-1} with no electrode rotation and the ECSA was estimated using 210 µC cm^{-2}_{Pt}.
3. In addition to the $H_{UPD,}$ the ECSA of the catalyst was also measured using the CO-stripping protocol; the 50 mol% CO/N_2 is bubbled into the electrolyte for 30 min. After bubbling, the working electrode was held at 0.08 V for 30 minutes; then, the electrolyte was purged with nitrogen for 30 minutes. Two consecutive cyclic voltammograms were recorded from 0.05 to 1.1 V at a scan rate of 20 mV s^{-1} and held at the final potential for 2 min to electrochemically strip all of the available CO molecules adsorbed on the Pt surface. The CO stripping charge was obtained from the difference in the charge between the two cyclic voltammograms (voltammograms similar to that shown in Fig S12). The ECSA was determined from the CO stripping charge by using the specific charge of 420 µC cm^{-2}_{pt}.

4. ORR I-V curve was measured during an anodic sweep from -0.01 to 1 V using a scan rate of 20 mV s^{-1} at different rotation speeds (400-1600 rpm) to evaluate the activity, and the kinetics of the catalyst in O_2 saturated 0.1 M $HClO_4$. The obtained LSV is corrected to baseline voltammetry in N_2 saturated condition, iR correction based on the uncompensated ohmic electrolyte measured via high-frequency AC impedance in N_2 saturated 0.1 M $HClO_4$, and correction for low local atmospheric pressure (88-90 kPa in Calgary, Canada). As described by Kocha et al. and Shinozaki et al. [8,10,27], the data were corrected to 100 kPa based on the reaction order concerning O_2 of 0.85. The electrochemical data was collected using SP-200 potentiostat (Biologic, France). This study referred to all the electrochemical potentials represented as the RHE reference electrode.

Results: The cyclic voltammograms for ionomer-free and ionomer-containing catalyst films for Pt/cPDA and Pt/C in nitrogen-saturated 0.1 M $HClO_4$ shown in Figures S5a and S5b, respectively, confirmed the presence of Pt catalyst. The Pt surface area was determined from the H_{upd} peak as well as CO stripping peaks (not shown). The linear sweep voltammetry of the catalyst films in the Oxygen-saturated electrolyte (Figure S6 a,b,c,d) demonstrated all the features of a good film, viz. limiting current plateau reached before 0.7 V (vs RHE) and limiting current of ~ 6 mA cm^{-2} at 1600 rpm. K-L plots further confirm the good quality of data. The higher activity of Pt/cPDA catalyst compared to commercial Pt/C (10 wt% TKK) is observed in the LSV comparison shown in Figure S7a. Similarly, the impact of ionomer poisoning can be observed from the comparison between ionomer-free and ionomer-containing catalysts for commercial Pt/C and our Pt/cPDA catalysts in Figures S7b and S7c, respectively. Since the mass loading may vary slightly between the catalyst films, it is sensible to compare specific activities (mA cm^{-2}_{Pt}) so as to normalize the activity with respect to electrochemically active surface area (cm^2_{Pt}). These comparisons are presented in Table S4, revealing – (i) higher specific activity for ionomer-free Pt/cPDA compared to ionomer-free commercial Pt/C (10 wt% Pt, TKK), (ii) lower suppression (24% for Pt/cPDA vs 42% for

Pt/C TKK) in activity due to ionomer at I:C mass ratio of 0.2. It was shown previously (Kocha et al., ref [29]) that activity suppression due to ionomer reaches an asymptotic level at an I:C ratio of 0.2 (Figure S8). The 42% suppression in the activity of Pt/C TKK (this study) due to ionomer at an I:C ratio of 0.2 is comparable to that reported by Kocha et al. for Pt supported on high surface area carbon (Pt/HSC), as shown in Figure S8. It is worth noting that 4 times higher diameter (~135 nm) of cPDA compared to ~30 nm diameter of Vulcan carbon (TKK) implies that the external surface area carbon support is 16 times lower for Pt/cPDA. Thus, at the same I:C ratio, theoretically, a thicker coating of ionomer and/or higher coverage of ionomer on Pt/cPDA can be expected. Despite this expected higher impact of ionomer on activity suppression for Pt/cPDA, the RDE results reveal lower suppression.

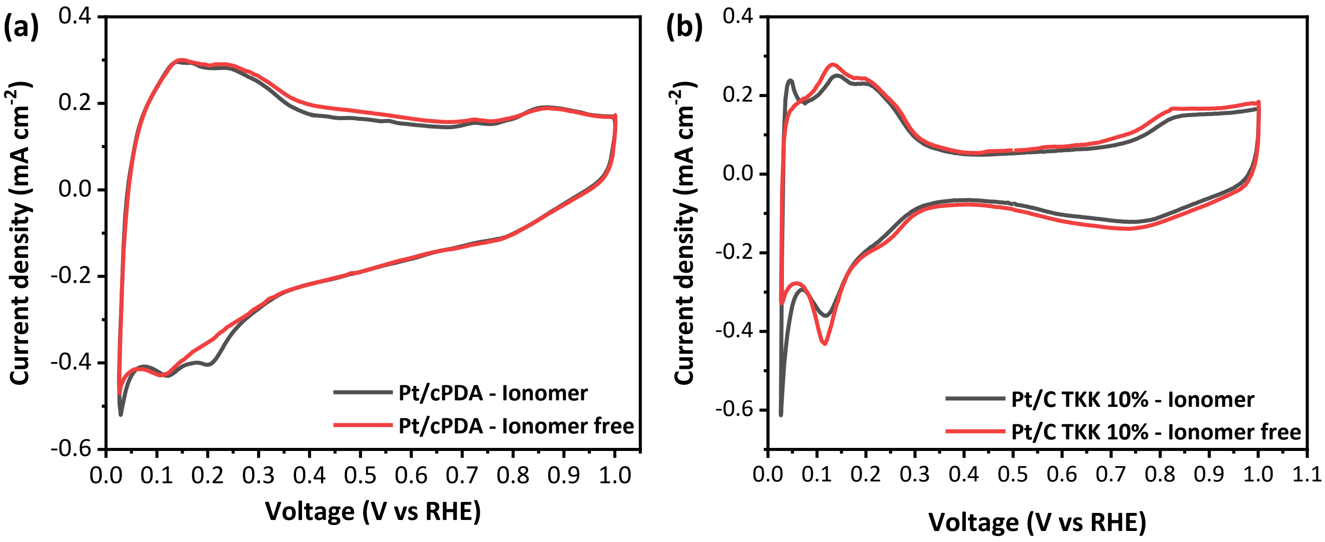

Fig. S5. Cyclic voltammogram of (a) Pt/cPDA catalysts (ionomer-free and with ionomer I:C = 0.2) and (b) Pt/C TKK catalysts (10 wt% - ionomer-free and with ionomer I:C=0.2) in N_2-saturated 0.1 M $HClO_4$ at 0 rpm, 23 °C, sweep rate - 20 mV s^{-1},. (RE - RHE; CE – Platinum).

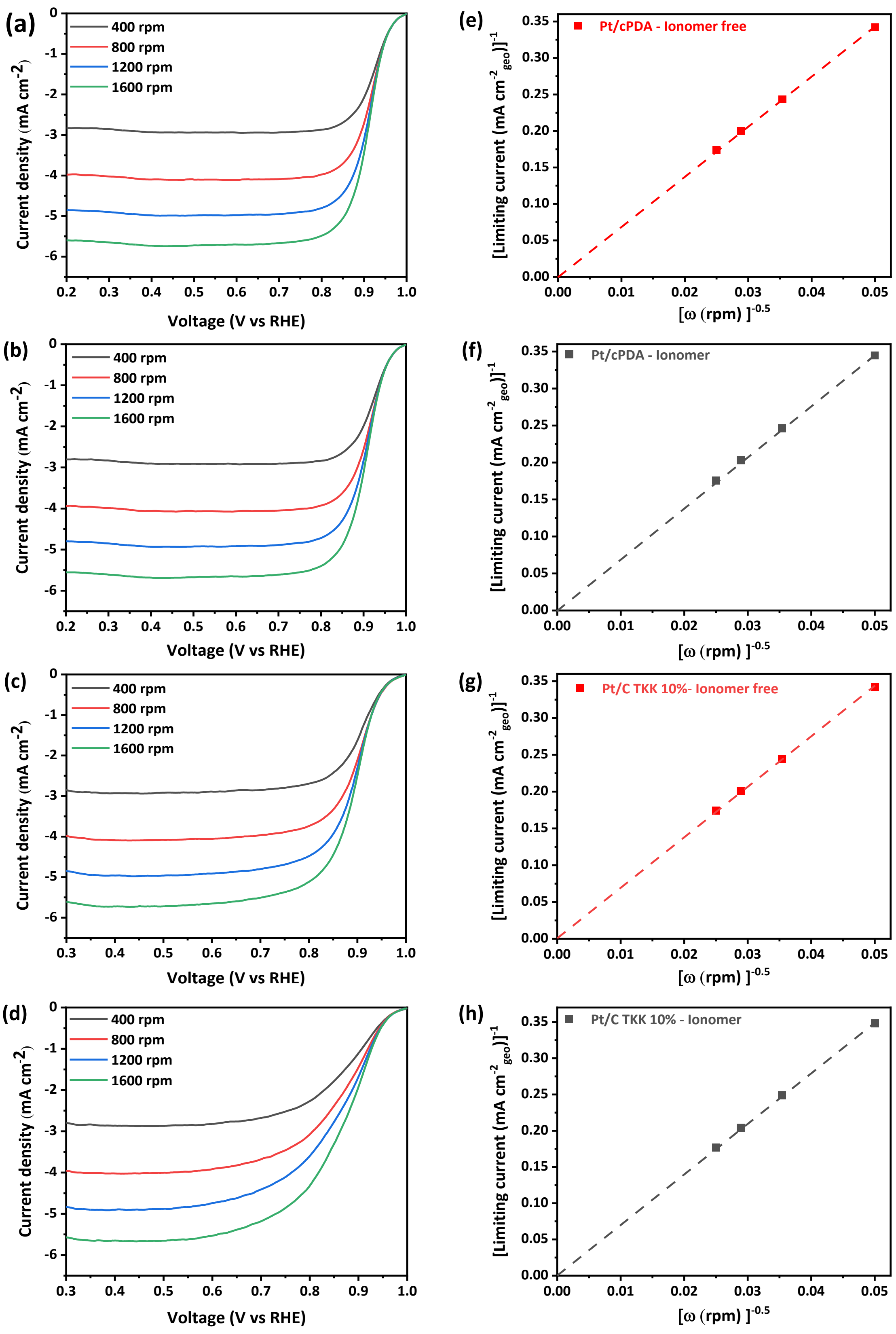
(a)
(b)
(c)
(d)
(e)
(f)
(g)
(h)
400 rpm
800 rpm
1200 rpm
1600 rpm
Current density (mA cm-2)
Voltage (V vs RHE)
[Limiting current (mA cm-2 geo)]-1
[ω (rpm)]-0.5
Pt/cPDA - Ionomer free
Pt/cPDA - Ionomer
Pt/C TKK 10%- Ionomer free
Pt/C TKK 10% - Ionomer

Fig. S6. Linear sweep voltammetry (LSV) catalyst at a different rotation speed in O_2 saturated 0.1 M $HClO_4$ at 23 °C, sweep rate - 20 mV s^{-1}. (RE - RHE; CE – Platinum) (a) Pt/cPDA catalyst (ionomer-free), (b) Pt/cPDA catalyst with ionomer I:C = 0.2, (c) Pt/C TKK catalyst (ionomer-free), (d) Pt/C TKK catalyst with ionomer I:C=0.2. The corresponding Koutecky-Levich plots (inverse limiting current versus inverse square root of rotational speed) at 0.4 V (vs RHE) for (e) Pt/cPDA catalyst (ionomer-free), (f) Pt/cPDA catalyst with ionomer I:C = 0.2, (g) Pt/C TKK catalysts (ionomer-free), (h) Pt/C TKK catalyst with ionomer I:C = 0.2.

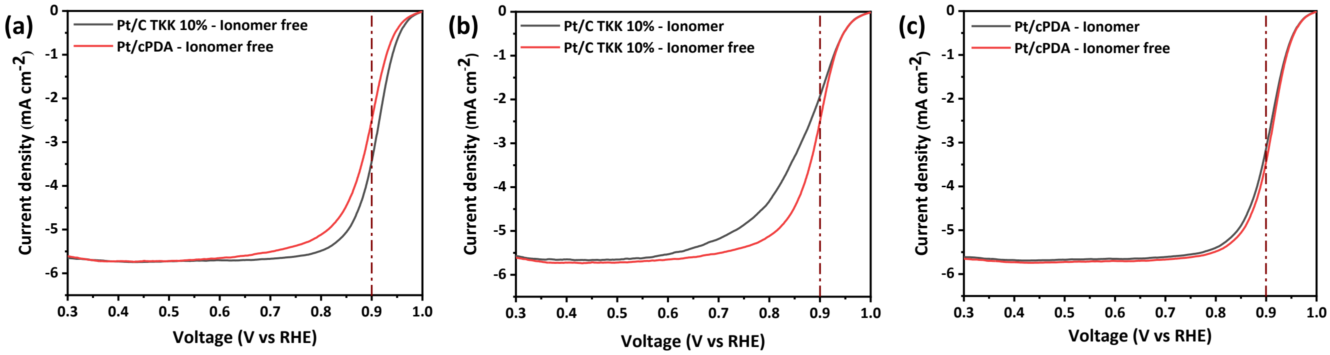

Fig. S7. Linear sweep voltammetry (LSV) comparison plot (a) for ionomer-free Pt/cPDA and ionomer-free Pt/C TKK (b) for ionomer-free and ionomer containing (I:C = 0.2) Pt/C TKK catalyst (c) for ionomer-free and ionomer containing (I:C = 0.2) Pt/cPDA catalyst. The rotation speed was 1600 rpm in O_2 saturated electrolyte 0.1 M $HClO_4$ at 23 °C, sweep rate - 20 mV s^{-1}. (RE - RHE; CE – Platinum).

Table S4: Summary of the ECSA and specific activity between Pt/cPDA (Ionomer and Ionomer free) and Pt/C TKK (Ionomer and Ionomer free)

Catalyst	ECSA ($m^2\ g_{Pt}^{-1}$)	Specific activity* ($mA\ cm^{-2}{}_{Pt}$)
Pt/cPDA-Ionomer free	107 ± 4	0.95 ± 0.07
Pt/cPDA-Ionomer (I:C ratio=0.2)	105 ± 7	0.72 ± 0.05
Pt/C TKK – Ionomer free	106 ± 11	0.43 ± 0.017
Pt/C TKK– Ionomer (I:C ratio=0.2)	102 ± 8	0.23 ± 0.016
Poly Pt	N/A (Roughness factor =1.73 ± 0.1)	1.77 ± 0.08

*The +/- represents deviations for 3-6 measurements – (a) made from at least two batches of ink for supported catalysts; the results on only the good films were considered (b) 3 repeat measurements of Poly Pt surface.

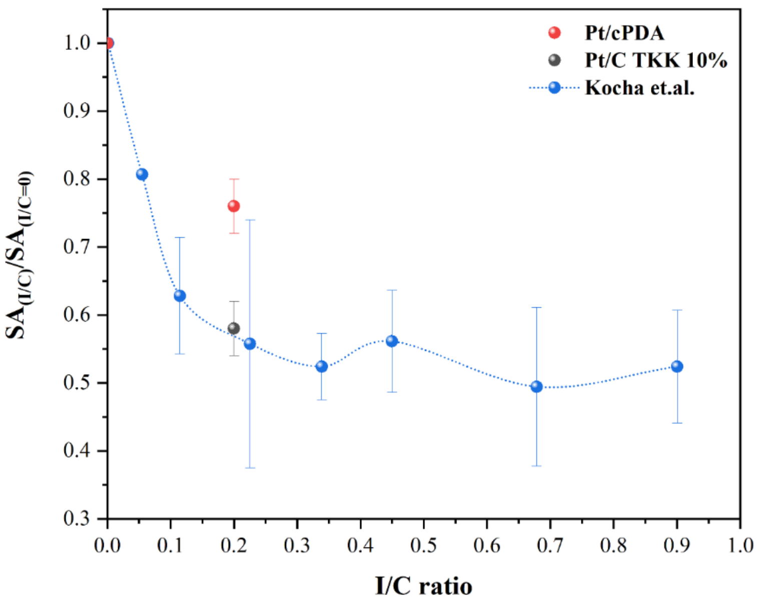

Fig. S8. Comparison of specific activity ratio (specific activity of ionomer-free catalyst: specific activity of ionomer containing catalyst) for Pt/cPDA(red), Pt/C TKK (black) and literature data by Kocha et al. (blue)(The literature data is digitized from ref[29]). *The error bar (red and blue) represents deviations for

3-6 measurements – made from at least two batches of ink for supported catalysts (Pt/cPDA and Pt/V(TKK)).

Fabrication of membrane electrode assembly (MEA)

In this study, the decal transfer method was used in order to prepare an MEA. First, the desired amount of IPA to achieve a solid to liquid ratio (S/L) of 0.18 and 20 wt% Nafion ionomer dispersion (EW 1100, Ion Power Inc.) .) was added to achieve an overall I/C mass ratio of 0.8 with catalyst (Pt/cPDA) in a 20 mL glass vial and dispersed ultrasonically using an ultrasonic bath for 20 minutes; both procedures were performed under the ice to avoid Pt degradation. For the commercial TKK 10% Pt/C catalyst, a similar ink preparation recipe described in reference[30] was used. Here, an ionomer to carbon ratio (I/C) of 0.8, a solid to liquid ratio (S/L) of 0.18 and a solvent mixture of 20% water/IPA were used to prepare the catalyst ink for the commercial TKK 10% Pt/C catalyst. Then, to further homogenize, the inks (both Pt/cPDA and TKK Pt/C) were magnetically stirred in a 20 mL glass vial containing 5 g of 5 mm diameter ZrO_2 beads for 24 h at room temperature (23° C) prior to its use. The catalyst layer was then coated onto a 75 µm thick PTFE substrate (McMaster-Carr, 8569K75) by using an automatic film coater (MSK AFA-II, MTI Corporation, USA) at a speed of 10 mm s^{-1}. During the coating, a wet film thickness of 100 µm was set. Subsequently, the catalyst-coated decal was air dried for 1 h followed by drying under vacuum for 12 h at 80 °C to evaporate residual solvents. CCM with an active area of 1 cm^2 (sub-framed and controlled by kapton window) was prepared by hot-pressing catalyst coated decal against 25 µm thick Nafion 211 (NRE-211, Fuel cell store, USA) membrane at 150 °C and 2 MPa pressure for 3 minutes with an applied force of 0.12 kN cm^{-2}. Pt loading in the prepared CCM was calculated gravimetrically by weighting the decals before and after the catalyst layer transfer. The thickness of Pt/cPDA and TKK Pt/C CLs was determined by a micrometre (Marathon digital micrometre, fisher scientific, USA) 9 ± 1 µm and 10 ± 1 µm, respectively.

In this study, three Pt/cPDA CCMs were prepared and tested separately for reproducibility; the error bar presented is from the average of results from three individual testings, except for the mass transport resistance which was repeated twice. The loading of Pt/cPDA and commercial Pt/C catalyst (10 wt% Pt/Vulcan, TKK) were (Pt/cPDA – 0.031, 0.033 and 0.036 mg_{Pt} cm^{-2}) and 0.058 mg_{Pt} cm^{-2}, respectively. After hot-pressing, a cell was assembled by sandwiching hotpressed CCM between two gas diffusion layers with a microporous layer (~ 230-240 µm, Toray, TGP-H-060; Fuel Cell Store, USA) guided by 175 µm thick PTFE gaskets to ensure around 25% compression of GDL. GDL dimensions were 1.5 cm x 1.5 cm on both sides. The area of the GDL was designed to be larger than the active area of the CCM to ensure independent control of compression of the catalyst layer and the GDL and also to avoide edge degradation. A torque of 30 in-lb was applied during the cell assembly in three steps (10-20-30 in-lb). Fuel cell hardware consisting of 50 cm^2 flow field with a serpentine channel (16 cm^2 channel area) was used (purchased from fuel cell technologies, USA). A photograph of the hardware, flow field and MEA is shown in Fig. S9.

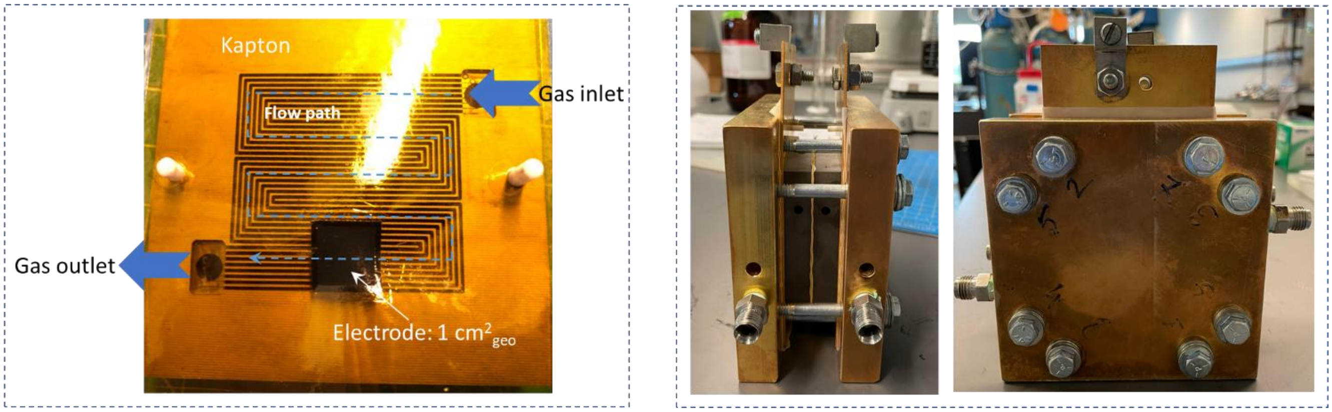

Fig. S9. Optical image of the flow-field design and MEA geometry with respect to the flow field (left) and front and side view of the used hardware for cell assembly (middle and right).

Fuel cell testing

Fuel cell testing with in-situ high-frequency resistance (HFR, for iR correction) measurement at each voltage were performed using a Biologic SP-200 potentiostat and a commercial 100 W, G20 Greenlight Innovation test station (Greenlight Innovation corp., Canada) in a high differential cell. The following sequence of events were followed and are described in detail below (Fig S10).

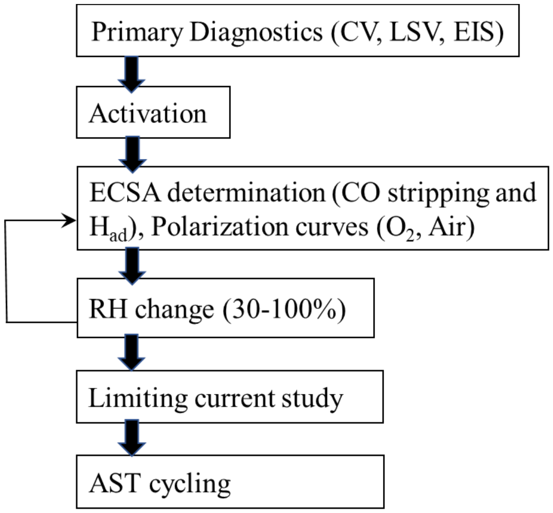

Fig. S10. Flow chart shows the sequence of testing for each cell.

Primary diagnostics (before activation): First, a series of primary diagnostic tests (H_{ad} CV, LSV and EIS) was performed to determine the electrochemical surface area (ECSA), H_2 crossover and series resistance or high-frequency resistance (HFR) of the cell for quality assurance of the assembled cell at 70°C, 100% RH, 140 kPa_{abs} in H_2/N_2 (0./0.2 NLPM). The reactants, i.e. compressed air (99.999%), carbon monoxide (99.5%), hydrogen, oxygen (99.999%), and nitrogen (99.999%), were obtained from Air Liquide, Canada. The anode (hydrogen electrode) served as the reference (RHE) and counter electrode (CE), while the cathode was the working electrode (WE). The impedance spectra were obtained at 0.4 V by sweeping frequencies in the range of 1 MHz to 1 Hz with an amplitude of 10 mV. The anode and working electrode (cathode) were fed with fully humidified hydrogen and nitrogen at the rate of 0.1 NLPM and 0.2 NLPM, respectively. Fig. S10 shows an overview of all the electrochemical tests performed in an MEA.

Activation Protocol: All cells were conditioned prior to testing to activate the MEA, hydrate the ionic network and remove possible contamination. The conditioning protocol used in this study is a combination of USFCC, DOE and recent work from the NREL (USA) group. The H_2 pumping from Ref [31], constant voltage hold at 0.6 V from USFCC[32] and potential cycling conditioning protocol (OCV to 0.6 V) similar to DOE[33] (OCV to 0.55 V) and Kabir et al. [34]. (OCV to 0.6V) were employed. The entire conditioning protocol is summarized in Table S5 and explained below.

Table S5. Summary of the conditioning protocol used in this study.

**Step #**	**Test**	**T_{cell} (ºC)**	**T_{hum} (ºC)**	**Flowrate An/Ca (NLPM)**	**Reactant gas An/Ca**	**Pressure (kPa_g)**	**Time (h)**
1	H_2 pumping	30	45	0.5/0.5	H_2/H_2	0	0.5
2	Flooding	70	80	0.1/0.2	H_2/N_2	50	8-12
3	0.6 V hold	70	70	0.3/0.5	H_2/Air	200	~12
4	Potential cycling (OCV-0.6V)/5x	70	70	0.3/0.5	H_2/Air	50	5 min hold at each potential

First, an H_2 pumping procedure was performed while the cell (both anode and cathode), anode humidifier and cathode humidifier temperature was set at 30 ºC, 45 ºC and 35 ºC, respectively. H_2 pumping was performed for 30 min by applying a current of 50-200 mA cm^{-2} while the resulting voltage was mildly negative. After that, the cell was purged with high flow N_2; then, the cell was supplied with over-humidified gas H_2/N_2 (0.1/0.2 NLPM) for around 8-12 h by setting the cell and humidifier temperature (both anode and cathode) at 70 ºC and 80 ºC, respectively to hydrate the membrane and ionomer network. This step results in a 10-20 mΩ-cm^2 reduction in the series resistance. Subsequently, the cell was purged again with a high flow of N_2 for 20 min to remove excess water, and the humidifier temperature was decreased to 70 ºC. Once the temperature is equilibrated, humidified H_2 and Air were introduced to the anode and cathode, respectively, and the back pressure was set at 200 kPa_g for both sides. When an open circuit voltage (OCV) of ~ 1.0 V was reached, a constant voltage hold of 0.6 V was applied to the cell and

current was drawn from the cell for ~ 12 h until the current stabilizes (± 5 mA cm^{-2}), similar to the one described in reference[35]. A typical current profile is provided in Fig. S11.

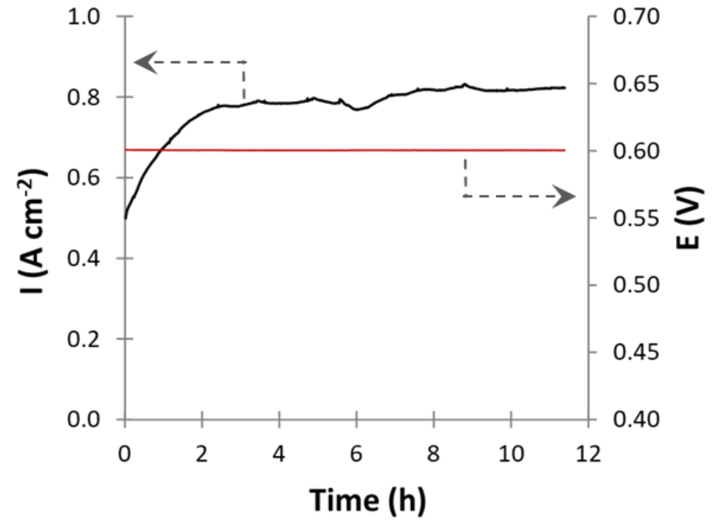

Fig. S11. Typical current profile during conditioning under constant voltage hold of 0.6 V at 70 ºC, 100% RH and 200 kPa_g pressure in H_2/Air (0.3/0.5 NLPM).

After the 0.6 V hold, the cell was purged again with a high flow of N_2 gas to remove the excess water. Finally, the cathode potential was cycled between OCV to 0.6 V by holding for 5 min at each potential at 70 ºC, 100% RH and 50 kPa_g pressure in H_2/Air (0.3/0.5 NLPM); this was repeated 5 times. A similar break-in protocol was also used by Kabir et al. [34]. The step 4 of the used activation protocol in this study is similar to the DOE protocol (OCV to 0.55 V). The cell was purged with a high flow rate of N_2 for 20 min after the conditioning to remove any excess moisture trapped in the cell.

ECSA determination by H_{ad} (MEA): The H_{ad} CV was performed at 70 ºC, 30-100% RH and 140 kPa_{abs} in H_2/N_2 (0.1/0.2 NLPM) by scanning the potential between 0.06 V and 1.2 V versus RHE at a scan rate of 200 mV s^{-1} for 10 cycles. ECSA was determined by integrating the H-adsorption peak of the hydrogen underpotential deposition (H_{ad}) region (0.07 V – 0.4 V) from the 10th cycle once the steady state was reached while subtracting capacitive currents. The area corresponding to H_{ad} of the CV plot was evaluated using the trapezoidal rule. Subsequently, the roughness factor (RF) and ECSA was calculated using equation (S2) and (S3), respectively.

$$Rf_{HAD}\ (cm^2_{Pt}/cm^2_{geo}) = \text{Integrated Area}\ (V.\ mA\ cm^{-2})/[0.210\ (mC\ cm^{-2}_{Pt}) \times \text{scan rate}\ (mV\ s^{-1}) \times 10^{-3} \times \text{geometrical area}\ (cm^2_{geo})] \quad \text{(S2)}$$

$$\text{ECSA} = Rf\ (cm^2_{Pt}\ cm^{-2}_{geo})/\text{Loading}\ ((mg_{Pt}\ cm^{-2}_{geo}) \quad \text{(S3)}$$

A value of 0.210 mC cm^{-2}_{Pt} was considered[36]. The double layer current was calculated by taking an average value of the charging and discharging currents in the range of 0.40 – 0.5 V. Crossover current was determined by performing LSV at a potential between 0.05 V and 0.6 V versus RHE at a scan rate of 5 mV s^{-1}.

ECSA determination by CO stripping: In addition to H_{ad} method, the ECSA of the MEAs was also measured following a similar CO-stripping protocol described by Takeshita et al. [37]. The cathode was first purged using 1 NLPM N_2 until OCV dropped below 150 mV, while the flow of the anode was kept constant at 0.2 NLPM H_2. Then, the cathode was cleaned by potential cycles between 0.115 and 0.94 V at the scan rate of 50 mV s^{-1} under N_2. CO stripping was performed by adsorbing CO (5% CO in N_2, 0.2 NLPM) for 20 min at 70 °C and ambient pressure while holding the cathode potential constant at 0.08 V. Subsequently; the residual CO was removed by purging N_2 for 20 min using a flow rate of 0.5 NLPM. After purging, the potential was swept from 0.115 V to 1 V with a scan rate of 20 mV s^{-1} and held at the final potential for 2 minutes to oxidatively strip all electrochemically available CO molecules adsorbed

on Pt catalysts under the flow of N_2 (0.2 NLPM). This sweep pattern was repeated twice in order to verify no residual CO on the electrode surface. The CO stripping charge was obtained from the difference in charge above the double layer potential region (0.4-0.6 V) between the first and second sweep-holds. The ECSA was determined from the CO stripping charge by using a specific charge of 420 μC $cm^{-2}{}_{Pt}$. A typical CO stripping voltammogram of TKK 10% Pt/C CL and Pt/cPDA CL is shown in Fig. S12. The CO stripping measurement was performed at varying relative humidity (30% RH to 100% RH) in order to calculate the dry proton accessibility (Fig. S13). A comparison of the ratio of ECSA determined by CO stripping and H_{ad} ($ECSA_{CO}/ECSA_{HAD}$) for Pt/cPDA and in-house TKK 10% Pt/C catalyst as a function of RH was shown in Fig. S14a. Also, for comparison, the dry proton accessibility ($ECSA_{CO}$ based) of 10% Pt/V (Padgett et al.[38]) at varying RH was plotted in Fig. S14a, and $ECSA_{CO}/ECSA_{HAD}$ of 10% Pt/V (Padgett et al. [38].) and 30% Pt/HSC (Garrick et al.[39]) of General Motors group was plotted in Fig. S14b.

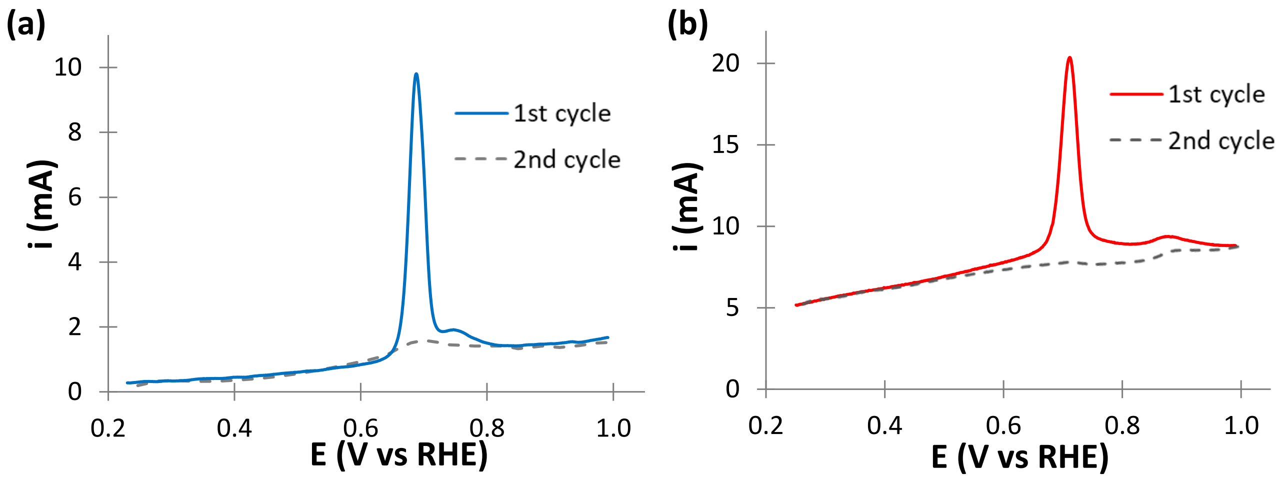

Fig. S12. Typical CO stripping voltammogram of (a) TKK 10% Pt/C CL and (b) Pt/cPDA CL at 70 ºC, 100% RH and ambient pressure at 20 mV s^{-1}.

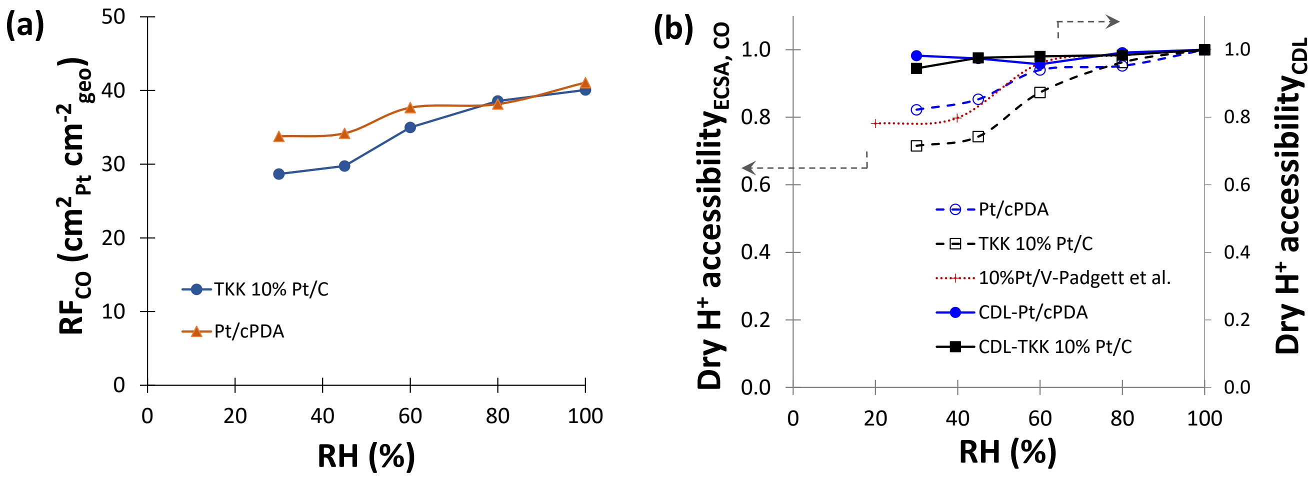

Fig. S13. (a) Roughness factor (RF, determined by CO stripping) comparison between Pt/cPDA and TKK 10% Pt/C catalyst as a function of RH, (b) dry proton (H^+) accessibility (estimated by $ECSA_{CO\ stripping}$ – open symbol, dashed line and C_{DL} – filled symbol, solid line) comparison between Pt/cPDA and in-house TKK 10% Pt/C catalyst and literature Pt utilization data of 10% Pt/V published by Padgett et. al..[38] *Condition: 70 ºC, Nafion EW1100, I/C – 0.8 (this work), 80 ºC, Nafion EW950, I/C – 0.95 (Padgett et. al.* [38]*)*

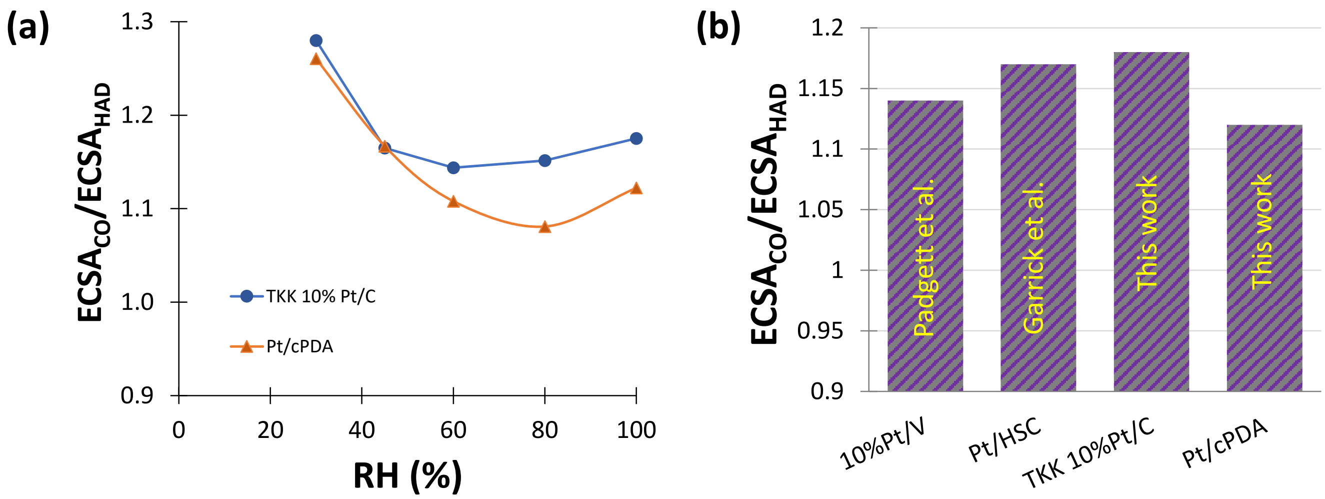

Fig. S14. (a) ECSA (CO stripping)/ECSA (H_{ad}) ($ECSA_{CO}/ECSA_{HAD)}$ ratio comparison (a) between Pt/cPDA and in-house TKK 10% Pt/C catalyst as a function of RH, (b) between in house Pt/cPDA and TKK 10% Pt/C catalyst, and literature 10% Pt/V (Padgett et al. [38]) and Pt/HSC catalysts (Garrick et. al.[39]) at 100% RH. *Condition: 70 ºC, Nafion EW1100, I/C – 0.8 (this work), 80 ºC, Nafion EW950, I/C – 0.95 (*Padgett et al.[38] and *Garrick et. al.*[39]*)*

Determination of kinetic parameters: In order to determine ORR kinetic parameters in MEA (mass activity, specific activity, and Tafel slopes), the current was recorded over 2 min by holding voltage at a specific voltage over the OCV to ~ 0.8 V range, where the voltage was decreased from OCV to ~ 0.8 V in ~ 30 mV decrements. The average value of current over the final 30 s were used to create an I-V plot. These measurements were part of the performance characterization in H_2/O_2 mode. Then, the voltage was corrected for iR (HFR) losses and normalized to the mass loading of Pt. The H_2 crossover was determined at the identical conditions as the polarization curve for which the corrections were applied, i.e., at the same temperature, pressure and RH; however, in H_2/N_2 configuration using LSV. The mass activity ($i_{m,0.9\ V}$) and specific activity ($i_{s,0.9\ V}$) were determined by interpolating to 0.9 V. Fig. S15a shows the mass loading normalized Tafel plots of Pt/cPDA catalyst at different RH and Pt free cPDA electrode. Fig. S15b presents the Tafel plots at both *70 ºC and 80 ºC.*

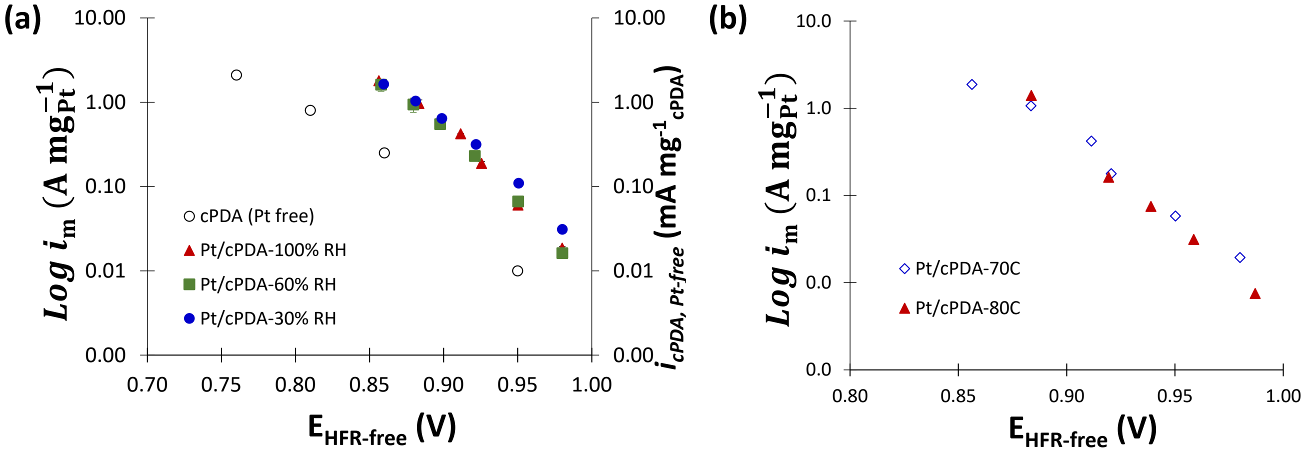

Fig. S15. Tafel plots of Pt/cPDA catalyst at (a) varying RH and Pt-free cPDA catalyst and (b) varying temperature (*70 ºC and 80 ºC*), *Condition: 70 ºC (a) and 140 kPa$_{abs}$ pressure, in H_2/O_2 (0.3/0.5 NLPM); voltage was corrected for iR (HFR) loss and current density was corrected for crossover loss.*

Table S6. Electrochemically determined geometric and kinetic parameters of Pt/cPDA and commercial Pt/C catalyst both in RDE and MEA condition.

Catalyst	*Geometric Parameters*			*Kinetic Parameters*		Reference
	ECSA ($m^2\ g_{Pt}^{-1}$)	RF ($cm_{Pt}^2\ cm^{-2}_{geo}$)	L_{Pt} ($\mu g_{Pt}\ cm_{geo}^{-2}$)	$i_{s,0.9V}$ ($mA\ cm_{Pt}^{-2}$)	$i_{m,0.9V}$ ($mA\ mg_{Pt}^{-1}$)	
Pt/cPDA-Ionomer free (RDE)	107 ± 4 (H_{ad})	–	~10	0.95 ± 0.07	944 ±10	This work
Pt/cPDA-Ionomer (RDE)	105 ± 7 (H_{ad})	-	~10	0.72 ± 0.05	737 ± 14	This work
Pt/cPDA (MEA)*	101 ± 5 (H_{ad})<br>113 (CO)	35 ± 2 (H_{ad})<br>41 (CO)	34 ± 2	0.632 ± 0.06	638 ± 68	This work
Pt/C TKK – Ionomer free (RDE)	106 ± 11 (H_{ad})	-	~10	0.43 ± 0.017	442 ± 14	This work
Pt/C TKK – Ionomer (RDE)	102 ± 8 (H_{ad})	-	~10	0.23 ± 0.016	249 ± 11	This work
Pt/C TKK (10 wt% Pt) (MEA)	70 (CO)	40 (CO)	58	0.147	103	This work

ECSA = Pt electrochemical surface area per unit mass of catalyst, RF = roughness factor of MEA working electrode (WE), L_{Pt} = WE Pt loading, $i_{s,0.9V}$ and $i_{m,0.9V}$ Pt specific and mass activity calculated at 0.9 V versus RHE, respectively

RDE condition: measured at a scan rate of 20 mV s^{-1} in 0.1 M $HClO_4$ at 23 ºC, ambient pressure (90 kPa); the obtained LSV is corrected to baseline voltammetry in N_2 saturated condition, iR correction based on the uncompensated ohmic electrolyte measured via high-frequency ac impedance in N_2 saturated 0.1M $HClO_4$, and pressure correction to 100 kPa – as described above in RDE section.

MEA condition: 70ºC, 100% RH, 140 kPa_{abs}, H_2/O_2 for activity and ECSA values were determined at 70 ºC, 100% RH, 140 kPa_{abs}, H_2/N_2 (for H_{ad}).

**the error represents the deviation from the average value measured for three independent samples.*

Polarization Curve: All polarization curves were determined in potentiostatic mode. The tests were recorded at desired operating conditions with H_2 and air or O_2 (0.3/0.5 NLPM) at 140 kPa_{abs} pressure. Both cathode and anode reactant gases were maintained at the same pressure during testing. Polarization plots were obtained from open circuit voltage (OCV) to around 0.1 V (until limiting current) potentiostatically by holding the voltage constant at each voltage for 3 minutes (steps of approximately 0.1 V after 0.9 V), and the resulting equilibrated current values were averaged over the final 30 s. In the case of pure O_2, polarization plots were obtained potentiostatically by recoding the I-V plot for kinetic parameter determination (as explained above, from OCV to around 0.8 V), followed by recording current from 0.8 V to around 0.1 V. HFR-corrected cell performance comparison between Pt/cPDA and TKK 10% Pt/C in both pure O_2 and Air at 70 ºC and 140 kPa_{abs} pressure (0.3/0.5 NLPM flow) was shown in Fig. S16, and uncorrected performance comparison was shown in the main body. Fig. S17 shows the uncorrected cell performance comparison and mass loading normalized performance comparison between Pt/cPDA and TKK10% Pt/C catalyst in H_2/O_2. Also, the mass loading normalized uncorrected performance comparison between Pt/cPDA and TKK10% Pt/C catalyst in Air was presented in Figure 5b of the main body. Fig. S18 shows the HFR-free mass loading normalized performance comparison between Pt/cPDA and TKK10% Pt/C in H_2/O_2 and H_2/Air. The HFR values used to perform all the iR corrections in the polarization curves recorded during the polarization curves are shown in Fig. S19. The higher HFR values (~ 100 mΩ-cm^2) of Pt/cPDA CL compared to that of Pt/V CL (~ 60 mΩ-cm^2) could be attributed to the higher interfacial contact resistance between the Pt/cPDA CL and MPL.

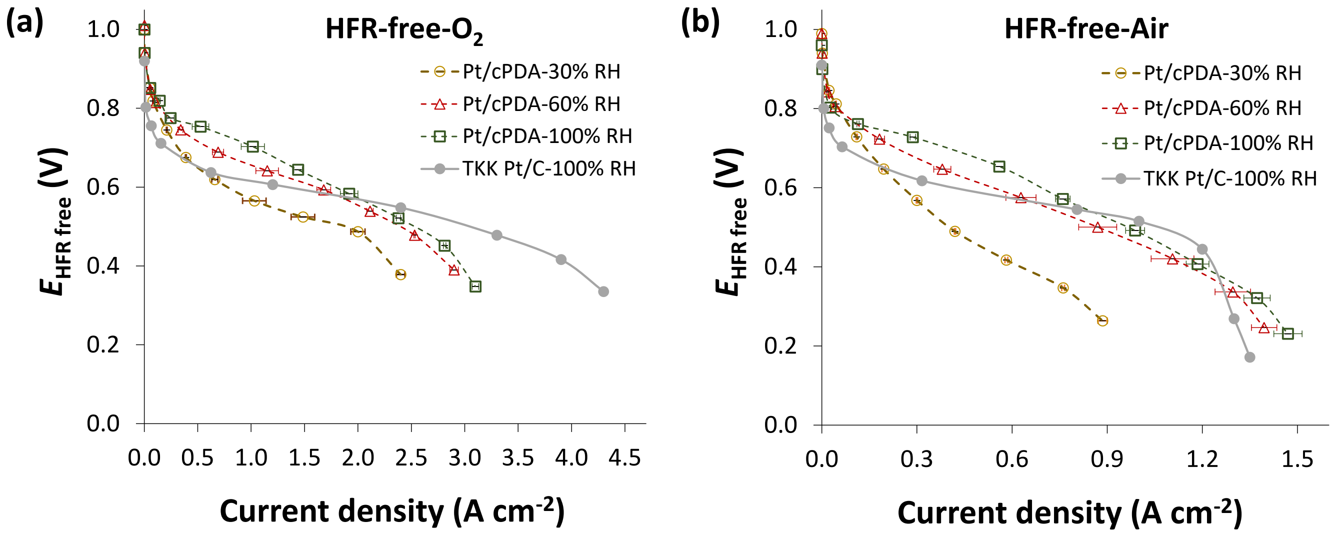

Fig. S16. HFR-free performance comparison between Pt/cPDA and TKK10% Pt/C catalyst : (a) in H_2/O_2, (b) H_2/Air. *Condition: 70 ºC and 140 kPa$_{abs}$ pressure (0.3/0.5 NLPM flow). Voltage was corrected for iR (HFR) loss, and current density was corrected for crossover loss. The error bar presented is from the average of results from three individual testings.*

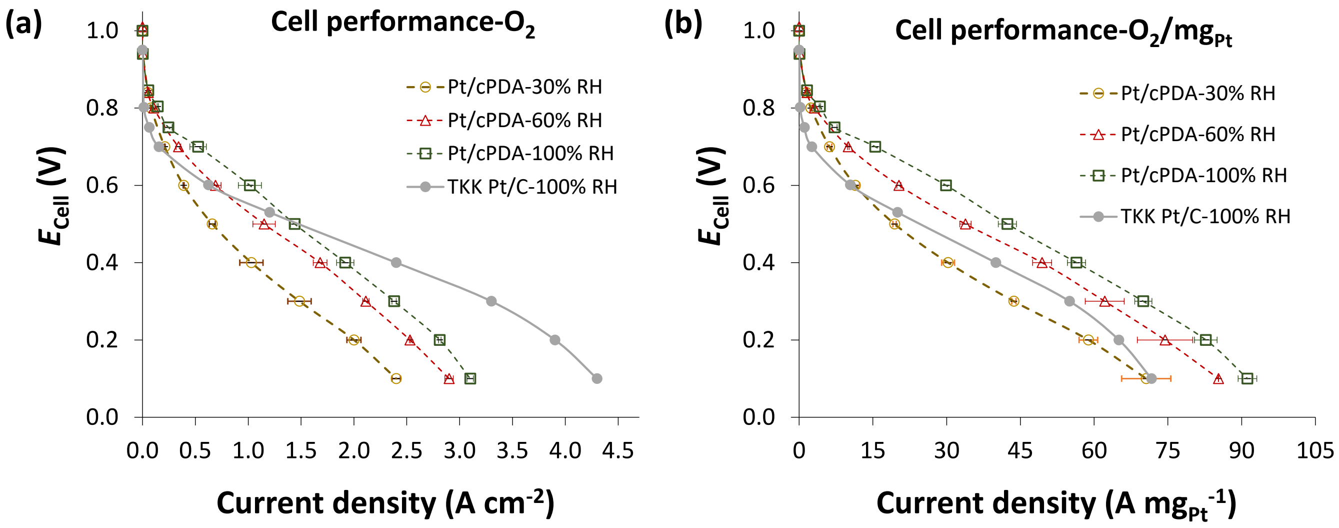

Fig. S17. Uncorrected (a) cell performance comparison and (b) mass loading normalized performance comparison between Pt/cPDA and TKK10% Pt/C catalyst in H_2/O_2. *Condition: 70 ºC and 140 kPa$_{abs}$*

pressure in (0.3/0.5 NLPM flow). The error bar presented is from the average of results from three individual testings.

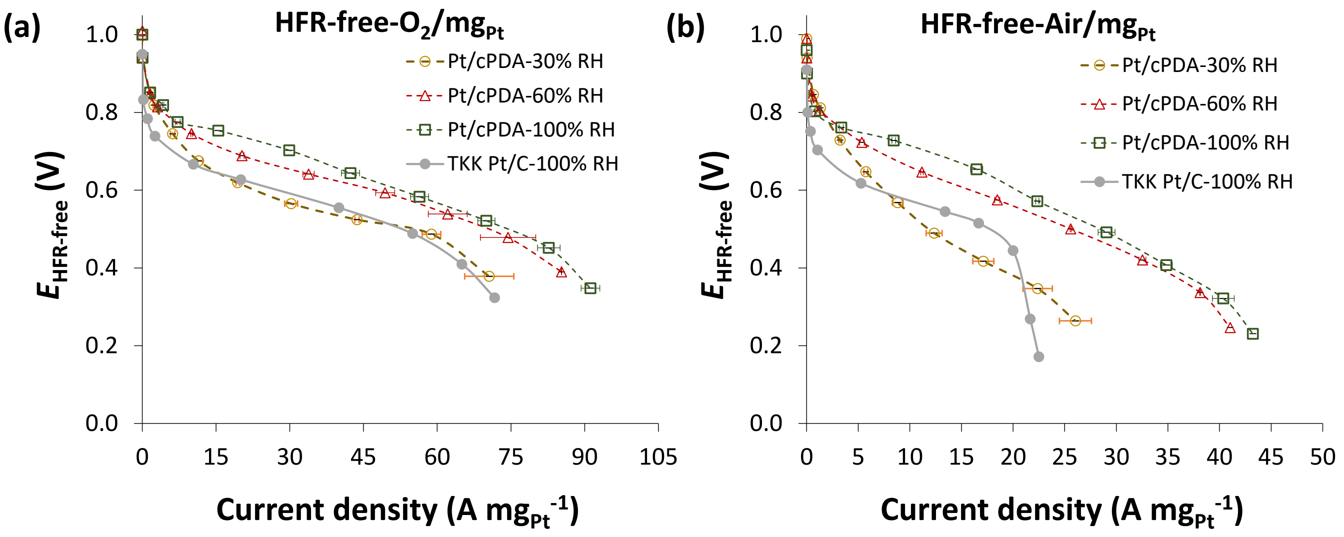

Fig. S18. HFR-free mass loading normalized performance comparison between Pt/cPDA and TKK10% Pt/C catalyst : (a) in H_2/O_2, (b) H_2/Air. *Condition: 70 ºC and 140 kPa$_{abs}$ pressure (0.3/0.5 NLPM flow). Voltage was corrected for iR (HFR) loss and current density was corrected for crossover loss. The error bar presented is from the average of results from three individual testings.*

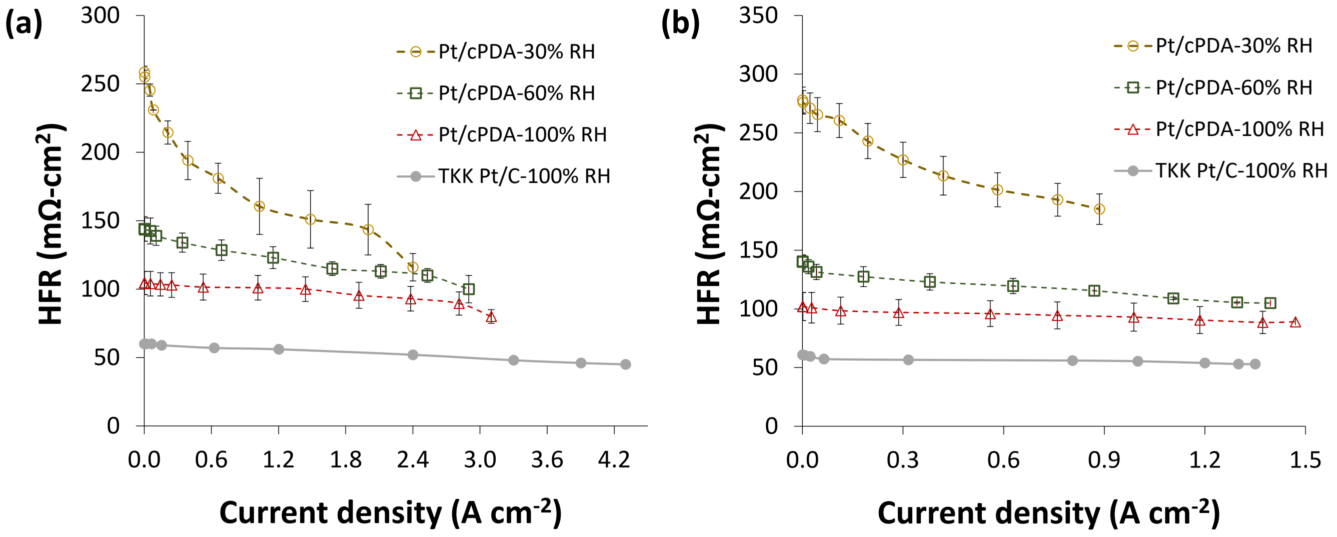

Fig. S19. (a) HFR values used to perform the iR corrections recorded during the polarization curves at different RH for Pt/cPDA and TKK Pt/C catalyst layer (100% RH): (a) in H_2/O_2, (b) H_2/Air. *Condition: 70 ºC and 140 kPa$_{abs}$ pressure in (0.3/0.5 NLPM flow). The error bar presented is from the average of results from three individual testings.*

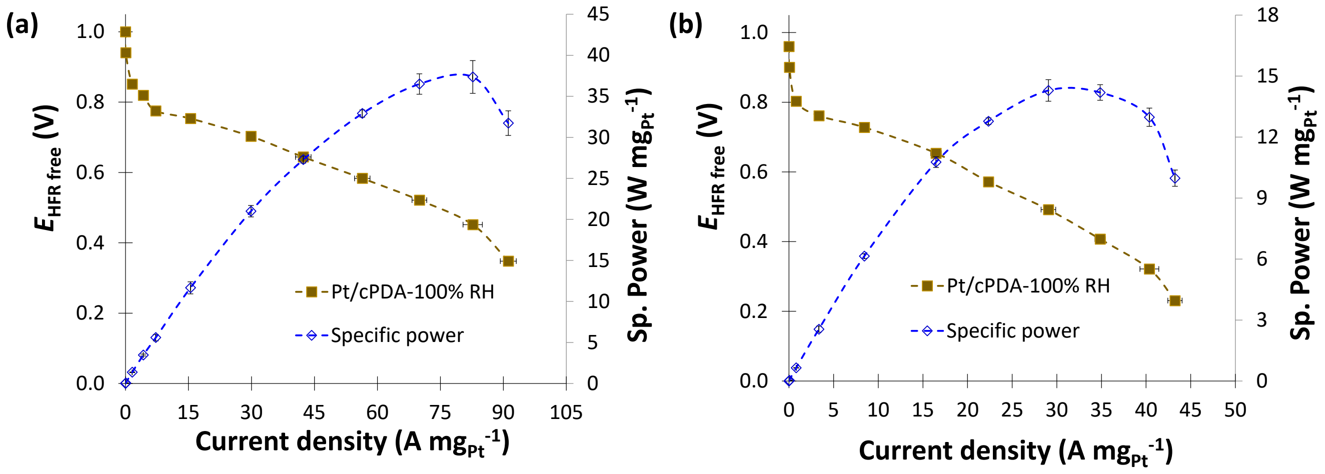

Fig. S20. Mass loading normalized polarization curve and specific power in (a) H_2/O_2 and (b) H_2/Air *(0.3/0.5 NLPM)* at 70 ºC, 100% RH and 140 kPa$_{abs}$; *Voltage was corrected for iR (HFR) loss and current density was corrected for crossover loss. The error bar presented is from the average of results from three individual testings.*

Limiting current study

The total mass transport resistance ($R_{O_2}^{total}$) was determined using the limiting current method developed by Baker *et al.* [40]. Limiting current (i_{lim}) measurements were performed at 70 °C and 80% RH. Differential flow conditions (0.3 NLPM of H_2 and 0.5 NLPM of O_2:N_2 mixtures) were used. The dry mole fraction of oxygen in the cathode is altered from 2 to 6% O_2 in N_2 using two external mass flow controllers. Typical limiting current profiles of Pt/cPDA CL in the potential range between 0.4 and 0.1 V at an O_2 concentration of 2 – 6% were shown in Fig. S21a (the marked regions indicate the limiting current values). In order to determine dry ($R_{O_2}^{total}$), limiting currents with an O_2 mole fraction of 2 – 6% were measured at four different total pressures (50, 100, 150 and 200 kPa_g). ($R_{O_2}^{total}$) was then plotted against total pressure and pressure-independent R_{O2} ($R_{O2}^{P,ind}$) was estimated from the Y-intercept of that plot. Fig. S21c and S21d show the ($R_{O_2}^{total}$) plots as a function of total pressure for Pt/cPDA and TKK 10% Pt/C CL, and at different RF of Pt/cPDA. Fig. S21c and S21d were used to estimate the ($R_{O2}^{P,ind}$) values. Fig. S21e illustrates a comparison of $R_{O2}^{P,ind}$ as a function of inverse of RF between the Pt/cPDA and literature obtained Pt/C based CLs data[41].

$$R_{O_2}^{total} = \frac{4F(C_{O_2,channel} - C_{O_2,Pt})}{i_{lim}} \approx \frac{4F \times C_{O_2,channel}}{i_{lim}} \qquad \text{(S4)}$$

$$R_{O_2}^{total} = R_{O_2}^{P,dep} + R_{O_2}^{P,ind} \qquad \text{(S5)}$$

$$R_{O_2}^{P,ind} = R_{O_2}^{Knudsen} + \frac{R_{O_2}^{local,Pt/I}}{r.f.} \qquad \text{(S6)}$$

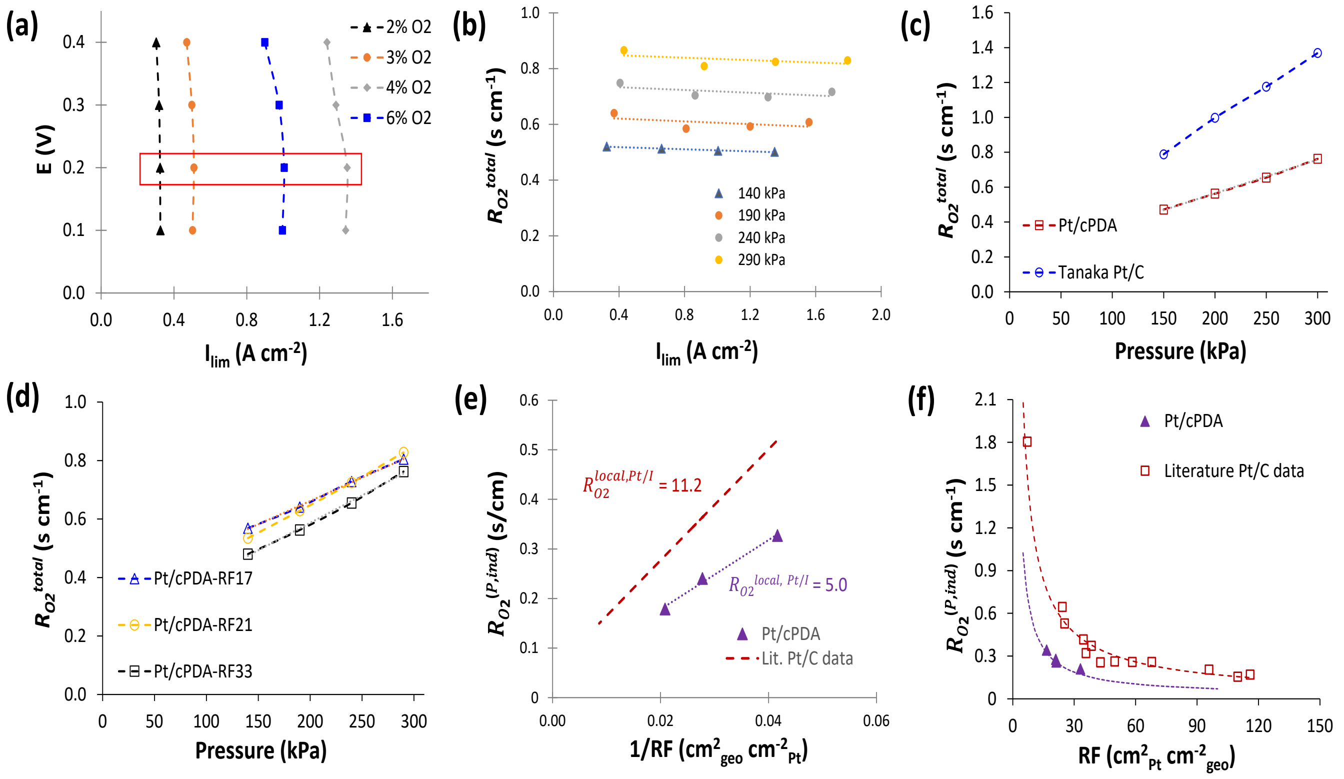

Fig. S21. (a) Limiting current profiles of Pt/cPDA CL in the potential range between 0.4 and 0.1 V at O_2 concentration of 2%, 3%, 4% and 6%, the marked area indicates the limiting current values, (b) $R_{O_2}^{total}$ estimated from different O_2 concentrations (2-6 %) as a function of limiting current of Pt/cPDA CL, (c) $R_{O_2}^{total}$ comparison as a function of total pressure between Pt/cPDA and TKK Pt/C catalyst (TKK10% Pt), (d) $R_{O_2}^{total}$ comparison as a function of total pressure at different RF of Pt/cPDA, (e) $R_{O_2}^{P,ind}$ comparison between Pt/cPDA and literature data of Pt/C form ref [41] as a function of inverse of roughness factor (RF). (e) $R_{O2}{}^{P,ind}$ comparison between Pt/cPDA and other Pt based catalysts as a function of RF. (The literature data is digitized from ref [41]). The O_2 transport data at different RF was collected during the AST degradation (at RF 33, RF 21 and RF 17). *Condition: 70 ºC and 80% RH in (0.3/0.5 NLPM flow).*

Accelerated Stress Test (AST) protocol

The square-wave catalyst AST protocol[42] was used in this work to study the durability of Pt catalyst, with the only exception that the temperature was 70 °C instead of 80 °C. It consisted of potential cycling between 0.6 V and 0.95 V by holding the voltage at each potential for 3s for 30,000 cycles. It was conducted in H_2(anode)/N_2(cathode) at 0.2/0.2 NLPM at 70 ºC, 100% RH and atmospheric pressure. The catalytic mass activity and polarization curves were measured at the beginning (BOL) and EOL (after 30000 AST cycles). Electrochemical surface area (ECSA) was measured after every 2000 AST cycles by H_{ad} and CO stripping method. Fig. S22a shows the HFR-free polarization curves comparison at BOL and after 30000 AST cycles in H_2/Air for Pt/cPDA catalyst at 70 ºC, 100% RH and 140 kPa_{abs}. Tafel plot comparison at the BOL and after 30000 AST (EOL) cycles is illustrated in Fig. S22b.

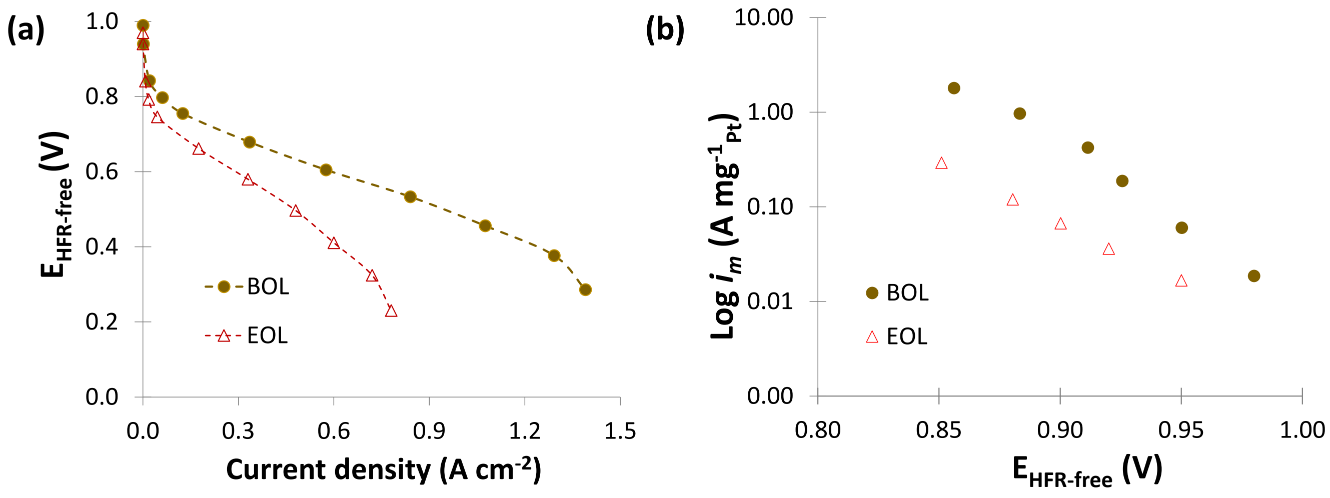

Fig. S22. HFR-free performance and Tafel plot comparison between BOL and EOL (after 30000 AST cycles) at 70 ºC, 100% RH and 140 kPa_{abs} in (a) H_2/Air and (b) H_2/O_2; *Voltage was corrected for iR (HFR) loss and current density was corrected for crossover loss.*

Discussion on transport resistance

The total O_2 transport resistance is a combination of pressure-dependent ($R_{O_2}^{P,dep}$) and pressure-independent ($R_{O_2}^{P,ind}$) resistances as shown in equation S5. The $R_{O_2}^{P,dep}$ (molecular diffusion) part depends on the fuel cell hardware, i.e., the flow-field channel geometry, the location of MEA on the flow-field, and the GDL types. In the serpentine flow-field, under the rib, convection can occur between some channels where the pressure difference between two channels is large. This effect has shown to be significant in single-serpentine channel compared to three-parallel channel[43]. In five parallel channel configurations, such as that employed in our study, the contribution of under-the-rib convection to enhancing the overall oxygen transport would be even smaller. If present, the under-the-rib convection will manifest in lowering the pressure-dependent oxygen transport resistance component of the overall transport resistance. However, the Knudsen diffusion and $R_{O_2}^{P,ind}$ will not be affected by the convective transport. Caution must be exercised in comparing total oxygen transport resistance from different studies by examining if the configuration employed may lead to under-the-rib convection.

On the other hand, $R_{O_2}^{P,ind}$ counterpart is composed of restrictive Knudsen diffusion resistance, which is mainly governed by the CL and MPL pore structure, and the resistance of O_2 transport through the Pt–ionomer–water interface, also known as local O_2-transport resistance. The local O_2-transport resistance is known to be affected by the equivalent weight of the ionomer used[44,45], I/C ratio (ionomer thickness)[30], uniformity or distribution of ionomer over the catalyst surface[46,47], types of carbon support[48], size, location and dispersion of the Pt particles[49]. On the other hand, the Knudsen diffusion resistance originated from CL depends on CL microstructure and pore size[50].

To the best of our knowledge, our Pt/cPDA CL exhibited the lowest $R_{O_2}^{P,ind}$ among the reported values in literature, as shown in Fig. 4e in the main body of the manuscript. The comparison is not so straightforward

but rather complex, as the aforementioned factors affect the $R_{O_2}^{P,ind}$. For example, the closest reported $R_{O_2}^{P,ind}$ was reported by Ott et al. around 0.23 s cm^{-1} at a higher loading of 0.1 mg$_{Pt}$ cm^{-2}. However, they have used a low EW 3M™ Dyneon™ PFSA ionomer with an I/C ratio of 0.65, 15% Pt/C catalyst and KB-based carbon support. Ono et al. reported a $R_{O_2}^{P,ind}$ of around 0.32 s cm^{-1} at a loading of 0.12 mg$_{Pt}$ cm^{-2}, where they have employed Nafion EW 1000 with an I/C ratio of 0.9, 15% Pt/C catalyst on KB carbon support. Among the compared values, only Owejan et al.[49] have used similar catalyst loading (0.025 mg$_{Pt}$ cm^{-2}) with similar Pt content of 10% Pt/V and ionomer (Nafion EW 1100); the I/C ratio was, however, a bit different (0.95) from this work. That exhibited a $R_{O_2}^{P,ind}$ of around 0.5 s cm^{-1}, almost 2 times higher than the Pt/cPDA catalyst layer.

Recent findings[44,45] suggest that the low EW ionomers exhibit higher local O2 transport resistance owing to higher amounts of adsorbed and/or absorbed water. On the contrary, some studies suggested that higher water content resulted in higher permeability of oxygen proton exchange membranes[51–53]. In addition to ionomer EW, the effective ionomer thickness, which is often dictated by the I/C ratio, also substantially affect the local O2 transport resistance. The recent findings from Gasteiger's group suggested that homogenous ionomer distribution and/or low I/C ratio, which eventually leads to low effective ionomer thickness, reduces the local transport resistance significantly[30]. Furthermore, location, distribution and dispersion of Pt particles, and even the Pt deposition method are also known to impact the local transport resistance[47,49]. Harzer and colleagues[47] postulated that the Pt particles located on the external surface of the carbon support, either controlled by the synthetic method or by the type of carbon support, exhibited lower oxygen transport resistance.

Table S7. Cathode and its subcomponents properties for the literature works used in this study to compare the pressure independent transport resistance ($R_{O_2}^{P,ind}$).

Ref*	Ionomer	Catalyst	I/C	Pt/C	GDL	Condition
Orfanidi (0.078)[30]	Asahi Kasei,EW700	Pt/V-NH_X	0.65	20%	Not mentioned	80 ºC, 70% RH, 170 kPa_{abs}
Harzer (0.063) [47]	Asahi Kasei,EW700	Pt/KB-$(NH_X)_{PO}$	0.65	20%	Not mentioned	80 ºC, 70% RH, 170 kPa_{abs}
Owejan (0.025) [49]	Nafion EW1100	Pt/V	0.95	10% + C (dilution)	Mitsubishi Rayon Co. U-105 with MPL	80 ºC, 80% RH, 150 kPa_{abs}
Ono (0.12)[45]	Nafion EW1000	Pt/V TKK	0.9	50%	TGP-H060	80 ºC, 90% RH, 101 kPa_{abs}
Ott (0.1)[46]	3M™ Dyneon™ PFSA (low EW)	Pt/KB	0.65	15%	SGL 29BC	80 ºC, 100% RH, 170 kPa_{abs}

*Values inside the bracket indicate Pt loadings in mg_{Pt} cm^{-2}

References

1. Ai, K., Liu, Y., Ruan, C., Lu, L. & Lu, G. Sp2 C-dominant N-doped carbon sub-micrometer spheres with a tunable size: A versatile platform for highly efficient oxygen-reduction catalysts. *Adv. Mater.* **25**, 998–1003 (2013).

2. VanBruinessen, A. & Karan, K. Development of Pt/CNT catalyst and transport-kinetic characterization of PEMFC catalyst layer. (Queen's University, 2009).

3. He, M., Zhou, S., Zhang, J., Liu, Z. & Robinson, C. CVD growth of N-doped carbon nanotubes on silicon substrates and its mechanism. *J. Phys. Chem. B* **109**, 9275–9279 (2005).

4. Soboleva, T. *et al.* On the micro-, meso-, and macroporous structures of polymer electrolyte membrane fuel cell catalyst layers. *ACS Appl. Mater. Interfaces* **2**, 375–384 (2010).

5. Meier, J. C. *et al.* Design criteria for stable Pt/C fuel cell catalysts. *Beilstein J. Nanotechnol.* **5**, 44–67 (2014).

6. Takahashi, I. & Kocha, S. S. Examination of the activity and durability of PEMFC catalysts in liquid electrolytes. *J. Power Sources* **195**, 6312–6322 (2010).

7. Kocha, S. S. *et al.* Best Practices and Testing Protocols for Benchmarking ORR Activities of Fuel Cell Electrocatalysts Using Rotating Disk Electrode. *Electrocatalysis* **8**, 366–374 (2017).

8. Shinozaki, K., Zack, J. W., Pylypenko, S., Pivovar, B. S. & Kocha, S. S. Oxygen Reduction Reaction Measurements on Platinum Electrocatalysts Utilizing Rotating Disk Electrode Technique. *J. Electrochem. Soc.* **162**, F1384–F1396 (2015).

9. Garsany, Y., Baturina, O. A., Swider-Lyons, K. E. & Kocha, S. S. Experimental methods for quantifying the activity of platinum electrocatalysts for the oxygen reduction reaction. *Anal.*

Chem. **82**, 6321–6328 (2010).

10. Shinozaki, K., Pivovar, B. S. & Kocha, S. S. Enhanced Oxygen Reduction Activity on Pt/C for Nafion-free, Thin, Uniform Films in Rotating Disk Electrode Studies. *ECS Trans.* **58**, 15–26 (2013).

11. Shinozaki, K., Zack, J. W., Richards, R. M., Pivovar, B. S. & Kocha, S. S. Oxygen Reduction Reaction Measurements on Platinum Electrocatalysts Utilizing Rotating Disk Electrode Technique. *J. Electrochem. Soc.* **162**, F1144–F1158 (2015).

12. Sugawara, S. *et al.* Performance decay of proton-exchange membrane fuel cells under open circuit conditions induced by membrane decomposition. *J. Power Sources* **187**, 324–331 (2009).

13. Markovic, N., Hanson, M., McDougall, G. & Yeager, E. The effects of anions on hydrogen electrosorption on platinum single-crystal electrodes. *J. Electroanal. Chem.* **214**, 555–566 (1986).

14. Molodkina, E. B. *et al.* Electroreduction of nitrate ions on Pt(1 1 1) electrodes modified by copper adatoms. *Electrochim. Acta* **56**, 154–165 (2010).

15. Schmidt, T. J., Paulus, U. A., Gasteiger, H. A. & Behm, R. J. The oxygen reduction reaction on a Pt/carbon fuel cell catalyst in the presence of chloride anions. *J. Electroanal. Chem.* **508**, 41–47 (2001).

16. Dima, G. E., Beltramo, G. L. & Koper, M. T. M. Nitrate reduction on single-crystal platinum electrodes. *Electrochim. Acta* **50**, 4318–4326 (2005).

17. Da Cunha, M. C. P. M., Weber, M. & Nart, F. C. On the adsorption and reduction of NO3- ions at Au and Pt electrodes studied by in situ FTIR spectroscopy. *J. Electroanal. Chem.* **414**, 163–170 (1996).

18. Subbaraman, R., Strmcnik, D., Stamenkovic, V. & Markovic, N. M. Three phase interfaces at electrified metal-solid electrolyte systems 1. study of the pt(hkl)-nafion interface. *J. Phys. Chem. C* **114**, 8414–8422 (2010).

19. Rima, F. R., Nakata, K., Shimazu, K. & Osawa, M. Surface-enhanced infrared absorption spectroscopic studies of adsorbed nitrate, nitric oxide, and related compounds. 3. Formation and reduction of adsorbed nitrite at a platinum electrode. *J. Phys. Chem. C* **114**, 6011–6018 (2010).

20. Pell, W. G., Zolfaghari, A. & Conway, B. E. Capacitance of the double-layer at polycrystalline Pt electrodes bearing a surface-oxide film. *J. Electroanal. Chem.* **532**, 13–23 (2002).

21. Dima, G. E., De Vooys, A. C. A. & Koper, M. T. M. Electrocatalytic reduction of nitrate at low concentration on coinage and transition-metal electrodes in acid solutions. *J. Electroanal. Chem.* **554–555**, 15–23 (2003).

22. Ahmed, M. *et al.* Unprecedented structural sensitivity toward average terrace width: Nafion adsorption at Pt{ hkl } electrodes. *J. Phys. Chem. C* **115**, 17020–17027 (2011).

23. Chu, D., Tryk, D., Gervasio, D. & Yeager, E. B. Examination of the ionomer/electrode interface using the ferric/ferrous redox couple. *J. Electroanal. Chem.* **272**, 277–284 (1989).

24. Genshaw, M. A., Damjanovic, A. & Bockris, J. O. M. The role of hydrogen peroxide in oxygen reduction at rhodium electrodes. *J. Phys. Chem.* **71**, 3722–3731 (1967).

25. Maruyama, J., Inaba, M., Katakura, K., Ogumi, Z. & Takehara, Z. I. Influence of Nafion® film on the kinetics of anodic hydrogen oxidation. *J. Electroanal. Chem.* **447**, 201–209 (1998).

26. Rodríguez-López, J., Minguzzi, A. & Bard, A. J. Reaction of various reductants with oxide films on Pt electrodes As studied by the surface interrogation mode of scanning electrochemical

microscopy (SI-SECM): Possible validity of a Marcus relationship. *J. Phys. Chem. C* **114**, 18645–18655 (2010).

27. Kocha, S. S., Garsany, Y. & Myers, D. *Testing Oxygen Reduction Reaction Activity with the Rotating Disc Electrode Technique*. *DOE Webinar* http://energy.gov/eere/fuelcells/downloads/testing-oxygen-reduction-reaction-activity-rotating-disc-electrode (2013).

28. Pollet, B. G. & Goh, J. T. E. The importance of ultrasonic parameters in the preparation of fuel cell catalyst inks. *Electrochim. Acta* **128**, 292–303 (2014).

29. Kocha, S. S., Zack, J. W., Alia, S. M., Neyerlin, K. C. & Pivovar, B. S. Influence of Ink Composition on the Electrochemical Properties of Pt/C Electrocatalysts. *ECS Trans.* **50**, 1475–1485 (2013).

30. Orfanidi, A. *et al.* The Key to High Performance Low Pt Loaded Electrodes. *J. Electrochem. Soc.* **164**, F418–F426 (2017).

31. Chunzhi He, Zhigang Qi, A. K. Electrochemical method to improve the performance of H2/air PEM fuel cells and direct methanol fuel cells. US Patent 6,730,424 B1 (2004).

32. US Fuel Cell Council. UFSCC single cell test protocol. (http://www.members.fchea.org/core/import/PDFs/Technical%20Resources/MatComp%20Single%20Cell%20Test%20Protocol%2005-014RevB.2%20071306.pdf (Accessed on 18th August 2022)

33. Balogun, E., Barnett, A. O. & Holdcroft, S. Cathode starvation as an accelerated conditioning procedure for perfluorosulfonic acid ionomer fuel cells. *J. Power Sources Adv.* **3**, 100012 (2020).

34. Kabir, S. *et al.* Elucidating the Dynamic Nature of Fuel Cell Electrodes as a Function of

Conditioning: An ex Situ Material Characterization and in Situ Electrochemical Diagnostic Study. *ACS Appl. Mater. Interfaces* (2019) doi:10.1021/acsami.9b11365.

35. Bezmalinović, D., Radošević, J. & Barbir, F. Initial conditioning of polymer eelectrolyte membrane fuel cell by temperature and potential cycling. *Acta Chim. Slov.* **62**, 83–87 (2015).

36. Woods, R. Hydrogen adsorption on platinum, iridium and rhodium electrodes at reduced temperatures and the determination of real surface area. *J. Electroanal. Chem.* **49**, 217–226 (1974).

37. Takeshita, T., Kamitaka, Y., Shinozaki, K., Kodama, K. & Morimoto, Y. Evaluation of ionomer coverage on Pt catalysts in polymer electrolyte membrane fuel cells by CO stripping voltammetry and its effect on oxygen reduction reaction activity. *J. Electroanal. Chem.* **871**, 114250 (2020).

38. Padgett, E. *et al.* Editors' Choice—Connecting Fuel Cell Catalyst Nanostructure and Accessibility Using Quantitative Cryo-STEM Tomography. *J. Electrochem. Soc.* **165**, F173–F180 (2018).

39. Garrick, T. R., Moylan, T. E., Carpenter, M. K. & Kongkanand, A. Editors' Choice—Electrochemically Active Surface Area Measurement of Aged Pt Alloy Catalysts in PEM Fuel Cells by CO Stripping. *J. Electrochem. Soc.* **164**, F55–F59 (2017).

40. Baker, D. R., Caulk, D. A., Neyerlin, K. C. & Murphy, M. W. Measurement of Oxygen Transport Resistance in PEM Fuel Cells by Limiting Current Methods. *J. Electrochem. Soc.* **156**, B991 (2009).

41. Kongkanand, A. & Mathias, M. F. The Priority and Challenge of High-Power Performance of Low-Platinum Proton-Exchange Membrane Fuel Cells. *J. Phys. Chem. Lett.* **7**, 1127–1137 (2016).

42. Stariha, S. *et al.* Recent Advances in Catalyst Accelerated Stress Tests for Polymer Electrolyte

Membrane Fuel Cells. *J. Electrochem. Soc.* **165**, F492–F501 (2018).

43. Wang, X. D. *et al.* Channel aspect ratio effect for serpentine proton exchange membrane fuel cell: Role of sub-rib convection. *J. Power Sources* **193**, 684–690 (2009).

44. Poojary, S., Islam, M. N., Shrivastava, U. N., Roberts, E. P. L. & Karan, K. Transport and electrochemical interface properties of ionomers in low-pt loading catalyst layers: Effect of ionomer equivalent weight and relative humidity. *Molecules* **25**, (2020).

45. Ono, Y., Ohma, A., Shinohara, K. & Fushinobu, K. Influence of Equivalent Weight of Ionomer on Local Oxygen Transport Resistance in Cathode Catalyst Layers. *J. Electrochem. Soc.* **160**, F779–F787 (2013).

46. Ott, S. *et al.* Ionomer distribution control in porous carbon-supported catalyst layers for high-power and low Pt-loaded proton exchange membrane fuel cells. *Nat. Mater.* **19**, 77–85 (2020).

47. Harzer, G. S., Orfanidi, A., El-Sayed, H., Madkikar, P. & Gasteiger, H. A. Tailoring Catalyst Morphology towards High Performance for Low Pt Loaded PEMFC Cathodes. *J. Electrochem. Soc.* **165**, F770–F779 (2018).

48. Ramaswamy, N., Gu, W., Ziegelbauer, J. M. & Kumaraguru, S. Carbon Support Microstructure Impact on High Current Density Transport Resistances in PEMFC Cathode. *J. Electrochem. Soc.* **167**, 064515 (2020).

49. Owejan, J. P., Owejan, J. E. & Gu, W. Impact of Platinum Loading and Catalyst Layer Structure on PEMFC Performance. *J. Electrochem. Soc.* **160**, F824–F833 (2013).

50. Inoue, G. & Kawase, M. Effect of porous structure of catalyst layer on effective oxygen diffusion coefficient in polymer electrolyte fuel cell. *J. Power Sources* **327**, 1–10 (2016).

51. Takamura, Y., Nakashima, E., Yamada, H., Tasaka, A. & Inaba, M. Effects of Temperature and Relative Humidity on Oxygen Permeation in Nafion and Sulfonated Poly(Arylene Ether Sulfone). *ECS Meet. Abstr.* **MA2008-02**, 905–905 (2008).

52. Borka, K. & Ekdunge, P. Oxygen and Hydrogen Permeation in Bulk and Recast Films. *Journal of Applied Electrochemistry* vol. 27 117–123 (2013).

53. Takaichi, S., Uchida, H. & Watanabe, M. Distribution profile of hydrogen and oxygen permeating in polymer electrolyte membrane measured by mixed potential. *Electrochem. commun.* **9**, 1975–1979 (2007).

54. Shinozaki, K. Personal communcations with Karan, K. (Date: Feb 09,2022)
