# Supplementary material for: Designing fuel cell catalyst support for superior catalytic activity and low mass-transport resistance
Source: Nat Commun. 2022 Oct 18;13:6157. doi: 10.1038/s41467-022-33892-8 (PMC9579166; doi:10.1038/s41467-022-33892-8)
Supplement: Supplementary file 3 — Description to Additional Supplementary Information [file 41467_2022_33892_MOESM3_ESM.pdf]

Description of Additional Supplementary Files

Video: 3D TEM of a single Pt/cPDA particle
